# Norepinephrine Protects against Methamphetamine Toxicity through β2-Adrenergic Receptors Promoting LC3 Compartmentalization

**DOI:** 10.3390/ijms22137232

**Published:** 2021-07-05

**Authors:** Gloria Lazzeri, Carla L. Busceti, Francesca Biagioni, Cinzia Fabrizi, Gabriele Morucci, Filippo S. Giorgi, Michela Ferrucci, Paola Lenzi, Stefano Puglisi-Allegra, Francesco Fornai

**Affiliations:** 1 Department of Translational Research and New Technologies in Medicine and Surgery, University of Pisa, via Roma 55, 56126 Pisa, Italy; gloria.lazzeri@unipi.it (G.L.); gabriele.morucci@unipi.it (G.M.); filippo.giorgi@unipi.it (F.S.G.); michela.ferrucci@unipi.it (M.F.); paola.lenzi@unipi.it (P.L.); 2 I.R.C.C.S. Neuromed, via Atinense 18, 86077 Pozzilli, Italy; carla.busceti@neuromed.it (C.L.B.); francesca.biagioni@neuromed.it (F.B.); stefano.puglisiallegra@neuromed.it (S.P.-A.); 3 Department of Anatomy, Histology, Forensic Medicine and Orthopedics, Sapienza University of Rome, via A. Borelli 50, 00161 Rome, Italy; cinzia.fabrizi@uniroma1.it

**Keywords:** PC12, autophagy, norepinephrine, methamphetamine, LC3, autophagy vacuoles, cell compartmentalization

## Abstract

Norepinephrine (NE) neurons and extracellular NE exert some protective effects against a variety of insults, including methamphetamine (Meth)-induced cell damage. The intimate mechanism of protection remains difficult to be analyzed in vivo. In fact, this may occur directly on target neurons or as the indirect consequence of NE-induced alterations in the activity of trans-synaptic loops. Therefore, to elude neuronal networks, which may contribute to these effects in vivo, the present study investigates whether NE still protects when directly applied to Meth-treated PC12 cells. Meth was selected based on its detrimental effects along various specific brain areas. The study shows that NE directly protects in vitro against Meth-induced cell damage. The present study indicates that such an effect fully depends on the activation of plasma membrane β2-adrenergic receptors (ARs). Evidence indicates that β2-ARs activation restores autophagy, which is impaired by Meth administration. This occurs via restoration of the autophagy flux and, as assessed by ultrastructural morphometry, by preventing the dissipation of microtubule-associated protein 1 light chain 3 (LC3) from autophagy vacuoles to the cytosol, which is produced instead during Meth toxicity. These findings may have an impact in a variety of degenerative conditions characterized by NE deficiency along with autophagy impairment.

## 1. Introduction

A bulk of studies indicate that the integrity of norepinephrine (NE) transmission in the brain is fundamental to provide neuroprotection. In fact, NE protects against neurotoxicity [1,2,3,4,5,6] and neurodegeneration [7,8], including Parkinson’s disease [1,9,10], Alzheimer’s disease [11,12], epileptic seizures [13,14,15,16,17,18,19], brain ischemia [20,21,22], and psychostimulants [2,6].

In this study, we specifically consider the protection of NE against methamphetamine (Meth)-induced toxicity as previously shown in vivo in the course of genetic and pharmacological manipulation of the NE system [2,6,23,24].

Although a protective role of NE is relevant, the mechanisms responsible for such an effect remain elusive. A number of studies hypothesize that in vivo complex neural networks may mediate the protective effects of NE, which would indirectly affect neuronal survival [4,24]. This is compatible with the sub-cortical origin of this neurotransmitter from a number of brainstem reticular nuclei [25,26], which project throughout wide areas of the forebrain affecting glia, vessels, and neurons [4,26]. On the other hand, NE may directly protect by specifically acting on target neurons. In order to test whether NE directly protects target cells in the present study, we used a model of neurotoxicity, which is well characterized and consists in administering in vitro Meth to PC12 cells [27,28,29]. In this model, the effects of NE were assessed by measuring the amount of cell loss and cell alterations. Once a direct protection was observed, the study proceeds to analyze whether NE needs to enter within PC12 cells or it rather protects by activating specific adrenergic receptors (ARs). Thus, specific NE uptake inhibitors (NETs), desmethylimipramine (DMI), as well as ARs agonist and antagonists were administered alone, or in combination with NE, (i) to disclose a protective role for intracellular NE and/or (ii) to decipher which receptor subtype(s) is involved. Once pharmacological studies led to identifying the putative receptor mediating NE-induced protection, pharmacological analysis was combined with specific RNA receptor silencing to validate findings. In the second part of the study, the subcellular mechanisms triggered by specific ARs activation were investigated in order to comprehend the intracellular events stimulated by NE, which leads to neuroprotection. Since Meth is known to alter PC12 cell viability by deranging the ubiquitous autophagy pathway, which is key in maintaining cell survival, autophagy was specifically investigated. In detail, a recent study shows that Meth toxicity in PC12 cells dissipates the specific autophagy protein LC3 from autophagy vacuoles [30,31]. Therefore, we analyzed whether NE prevents the dissipation of LC3 from autophagy vacuoles induced by Meth and whether it rescues the impairment of the autophagy flux induced by Meth [32]. In fact, when counteracting the autophagy impairment with classic autophagy activators, Meth-induced LC3 dissipation is prevented along with restoration of a normal autophagy flux, and a full protection against Meth toxicity is obtained [30]. In this part of the study, the stoichiometric compartmentalization of LC3 within autophagy vacuoles was investigated along with the progression of the autophagy flux. Meth was administered in combination with various doses of NE or ARs agonists and antagonists as well as NE uptake blockers. In addition, the expression of β2-ARs was silenced.

Due to a powerful and ubiquitous effect of the autophagy machinery in modulating cell damage [29,32,33,34,35,36], its potential involvement in NE-induced protection was not investigated so far, and it represents the key point of the present experimental work.

To sum up, the present study addresses the following core questions:

(i) whether NE directly protects against Meth-induced toxicity;
(ii) whether such an effect is achieved through specific plasma membrane ARs activation, or
(iii) whether NE needs to be taken up in the cell to exert protection;
(iv) whether NE-induced protection counteracts the derangement of specific autophagy compartments and autophagy flux induced by Meth.

## 2. Results

### 2.1. Pilot Dose–Response Curves on Cell Viability to Select Specific Doses of NE and Meth to Be Administered in the Body of the Study

**Figure 1:**
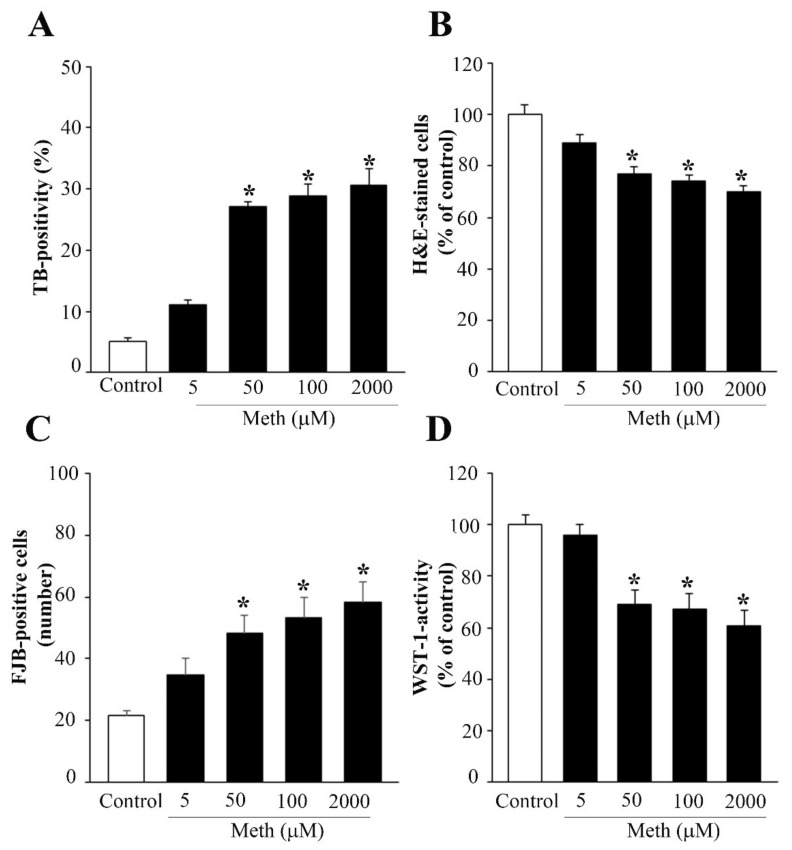
Dose–response curve for Meth-induced toxicity. A wide range of doses of Meth (5 μM, 50 μM, 100 μM, 1 mM, 2 mM) was administered to PC12 cells for 72 h. (**A**) Graph for TB-stained cells; (**B**) graph of H&E-stained cells; (**C**) graph of FJB-stained cells; (**D**) graph of WST-1 viability assay. While the lowest dose of Meth (5 μM) did not produce any decrease in cell viability compared with controls, Meth administration from the dose of 50 μM up to the dose of 2 mM produces a significant cell loss compared with controls, which was detected for each staining procedure. In detail, at 50 μM, the loss of cell viability already reached a plateau, since no further increase in toxicity was observed by using any procedure up to the dose of 2 mM. Data are given as the mean ± SEM of nine independent counts for TB and WST-1, and six independent counts for H&E and FJB. Inferential statistics was carried out with ANOVA with Scheffè’s post hoc analysis. (DF = 4). * *p* < 0.05 compared with controls.

Norepinephrine (NE), when applied at different concentrations within the nM range, which corresponds to the Ki for binding at various ARs (0.5 nM, 5 nM, 50 nM), does not affect PC12 cells viability assessed with different methods (Trypan Blue, TB, graph of Supplementary Figure S1A), hematoxylin and eosin (H&E, graph of Supplementary Figure S1B), and Fluoro-Jade B (FJB, graph of Supplementary Figure S1C). This pilot dose–response was carried out to assess the toxic dose of Meth to be used in these experiments. In fact, it is well known that depending on the experimental conditions, the toxicity induced by Meth in PC12 cells may significantly vary. In order to test these findings in a wide range of doses, in a few trials, we measured the effects of a dose of 5 μM, 50 μM, 100 μM, and up to 2 mM (the latter being well beyond the range of a Meth-dependent specific toxicity). A reliable consistency along various procedures was detected, showing that the dose of 50 μM Meth reaches a plateau of toxicity (roughly 25%) which is no further increased up to 2 mM (Figure 1).

In detail, the plateau for cell death was confirmed at the dose of 50 μM Meth along various procedures, TB (graph of Figure 1A), H&E (graph of Figure 1B), FJB (graph of Figure 1C), and WST-1 (graph of Figure 1D), caspase 3 immunofluorescence (Supplementary Figure S2), and transmission electron microscopy (TEM, Supplementary Figure S3), which is the gold standard to detect apoptosis and necrosis. These pilot experiments were crucial to assess which dose of Meth was appropriate to probe the protective effects of NE. It is remarkable that although the variability is documented across different studies of Meth-induced toxicity as reported above, this does not depend on variation in the cell cycle. In fact, in these preliminary experiments, similar results were obtained when PC12 cells were synchronized following 72 h of starvation. In this case, Meth was specifically administered when 78% of the cells were in the G0/G1 phase as shown by cytofluorometry (Supplementary Figures S4–S6); nonetheless, the amount of Meth-induced toxicity was similar. 

### 2.2. NE Does Protect against Meth-Induced PC12 Cell Death

**Figure 2:**
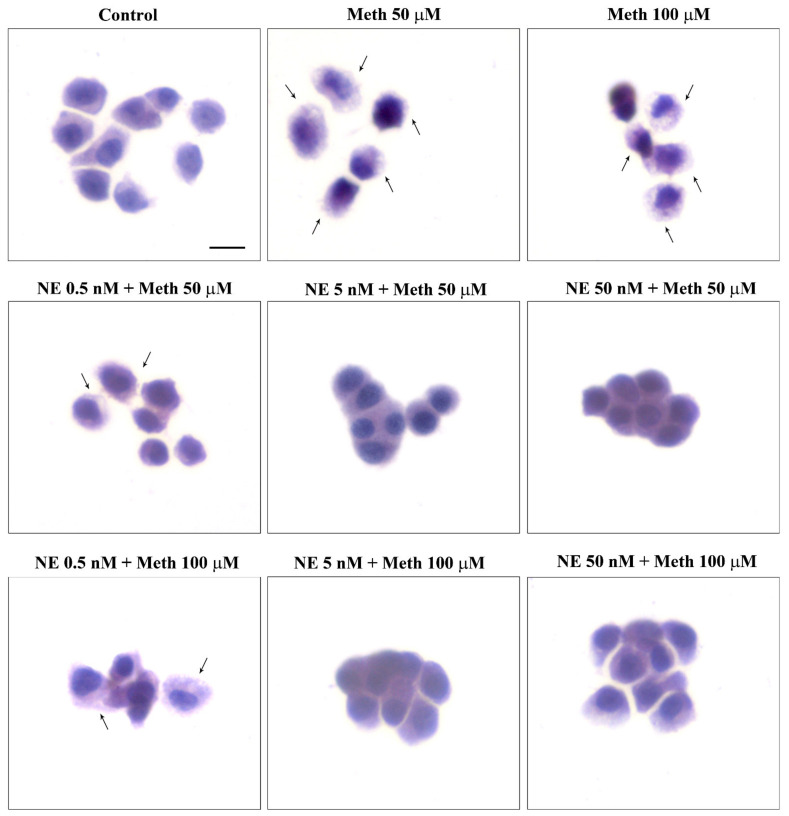
NE does protect against Meth toxicity (H&E staining). Representative pictures show NE protection against Meth-induced toxicity. The protective effects of NE are evident both at the dose of 5 nM and 50 nM administered to cells treated with Meth either at the dose of 50 μM or 100 μM. The toxic effects of Meth are detectable in spared cells as pale eosinophilic cytosol (arrows) where damaged cells are abnormal in size and shape. Scale bar = 8 μM.

**Figure 3:**
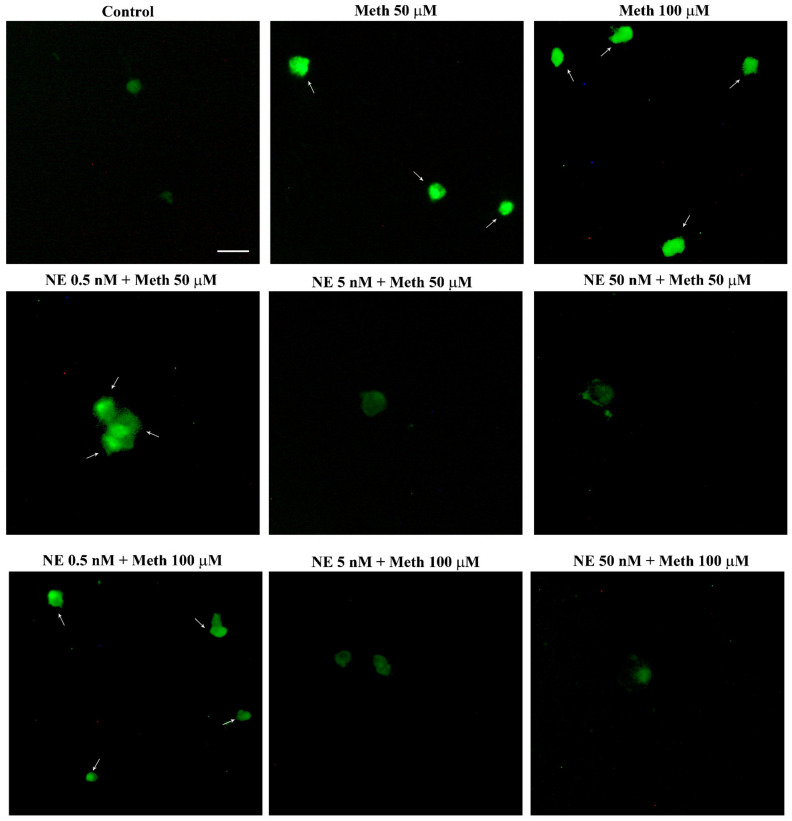
NE does protect against Meth toxicity (FJB staining). Representative pictures show NE protection against Meth-induced toxicity. The protective effects of NE are evident both at the dose of 5 nM and 50 nM, when administered to cells treated with Meth either at the dose of 50 μM or 100 μM. The toxicity induced by Meth is shown by intensely fluorescent spared cells (arrows). The protective effects of NE bring back the fluorescence to a pale signal as detected in Controls. Scale bar = 20 μM.

When assessing this key point of the study, the combined administration of NE and Meth was carried out for various Meth doses, each one already used in previous pilot experiments (Figure 1 and Supplementary Figure S1). In fact, we wish to assess a dose–response for NE-induced protection against Meth toxicity. As reported in the previous paragraph, the administration of NE alone, at different concentrations within the nM range (0.5 nM, 5 nM, and 50 nM) did not alter cell viability. Thus, we measured the protective potential of NE against toxicity produced by various doses of Meth. This experimental approach was applied here to check whether NE protection against Meth toxicity was achieved even when rising Meth concentration up to an amount, way in excess, to that needed to produce selective toxicity (Meth, 2 mM). As reported in graphs of Supplementary Figure S7 and representative Figure 2 and Figure 3, we found that even for the highest doses of Meth, NE, at the dose of 5 nM and 50 nM, fully protects. In contrast, the dose of NE 0.5 nM did not provide any protection against Meth toxicity (Figure 2 and Figure 3). Data concerning all the doses measured with all methods are reported in the graphs of Supplementary Figure S7A–D).

This indicates that a dose of 5 nM NE exerts full protection against Meth-induced toxicity. Therefore, a dose of 5 nM of NE, and a dose of 50 μM of Meth, were selected for the following experiments.

### 2.3. Effects of Various Agonists and Antagonists Acting on AR on Meth-Induced PC12 Cell Death

Once the protective effects of NE were established in a simple cell line, in the absence of brain circuitries, the key point was to decipher which AR known to be expressed on these cells [37] was involved in neuroprotection and whether NE may exert dual effects based on the concomitant activation of different ARs. Therefore, we assessed the effects of various α1-AR and β-AR agonists and antagonists in participating or fully producing protection, or even worsening Meth toxicity. As expected, phenylephrine (α1-AR agonist) at the standard dose of 10 μM alone did not affect cell mortality nor it did when co-administered with NE (graph in Supplementary Figure S8A for TB; graph in Supplementary Figure S8B for H&E; graph in Supplementary Figure S8C for FJB). Similarly, phenylephrine administration at the dose of 10 μM did not affect Meth-induced toxicity (same graphs of Supplementary Figure S8). Thus, α1-AR stimulation does not change Meth toxicity.

As expected, the administration of prazosin (a selective antagonist at α1-AR) at the dose of 15 μM neither reduced nor worsened Meth-induced cell death (graphs in Supplementary Figure S8A for TB; graph in Supplementary Figure S8B for H&E; graph in Supplementary Figure S8C for FJB). Similarly, prazosin did not modify the protective effects of NE against Meth toxicity (all the graphs in Supplementary Figure S8). 

The administration of isoproterenol (a β-AR non-selective agonist) at 400 μM does not induce any effect on baseline cell death (graphs of Supplementary Figure S9). However, it prevents cell death induced by Meth, either when administered alone, or in combination with NE. The amount of such a neuroprotective effect is similar to that induced by NE alone on Meth toxicity. This suggests that β-AR stimulation fully protects against Meth-induced toxicity and mediates the protection induced by NE.

This is confirmed by the non-selective β-AR antagonist propranolol. When co-administered with NE, propranolol (50 μM) prevents the protective effects induced by NE on Meth-induced cell death, while in cells concomitantly exposed to propranolol and Meth, the toxicity of Meth is not affected (Supplementary Figure S9). This latter finding suggests that the amount of cell death induced by Meth at such a dose cannot be further worsened neither by increasing the dose of Meth (as shown previously in Figure 1) nor by blocking the ongoing protections supposedly triggered by stimulating β-AR. In line with this, propranolol alone does not significantly increase baseline cell mortality compared with control cells, which suggests that baseline activity on β-AR does not protect these cells from their inherent, spontaneous vulnerability. As expected, the protective effects of isoproterenol on Meth-induced toxicity were occluded by the concomitant administration of propranolol (Supplementary Figure S9C).

Thus, β-AR fully protects against Meth-induced toxicity, thereby mimicking the effects of NE.

**Figure 4:**
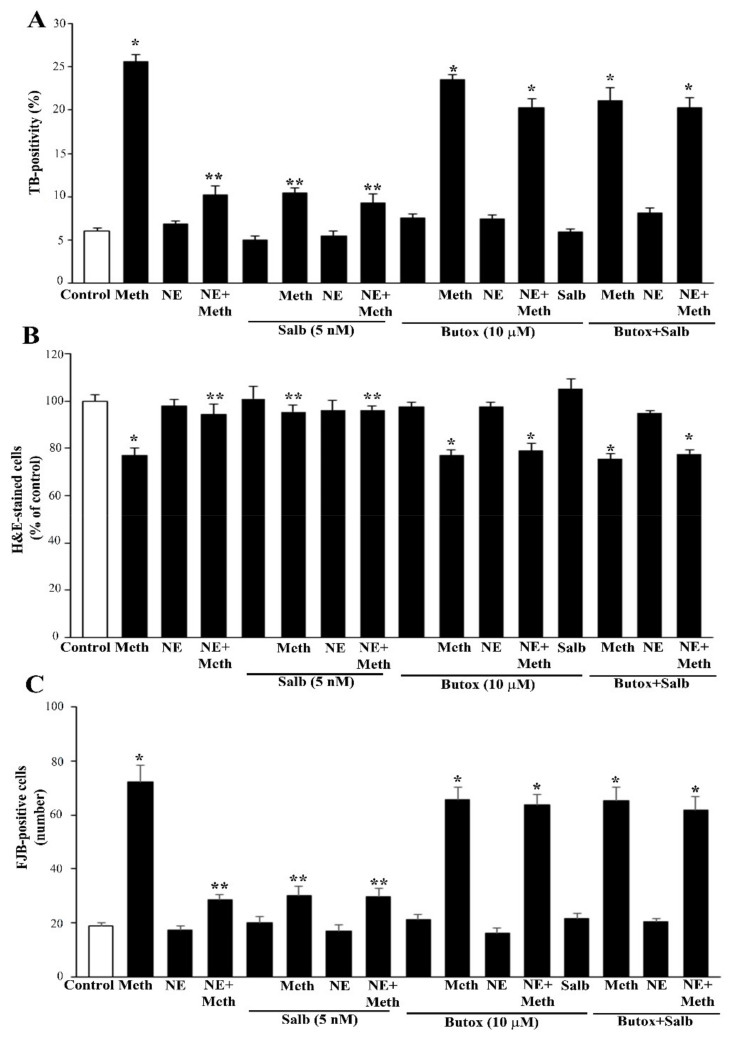
Pre-administration of the β2-AR agonist (salbutamol) protects against Meth toxicity. This effect is occluded by the β2-AR antagonist (butoxamine). The selective β2-AR agonist salbutamol (“Sal”, 5 μM) fully protects against the toxicity induced by Meth (50 μM). This protective effect is occluded in the presence of the selective β2-AR antagonist butoxamine (“Butox”, 10 μM). Butoxamine also fully prevents NE (5 nM)-induced protection against Meth toxicity, while it does not significantly modify Meth toxicity or spontaneous toxicity ongoing in control cells when administered alone. The effects on cell mortality of the β2-AR antagonist butoxamine (“Butox”) were assessed on naïve PC12 cells and in PC12 cells exposed to Meth (50 μM) and/or NE (5 nM). NE was used, either alone or in the group NE + Meth and “NE+Butox + Meth” starting 30 min before Meth administration; butoxamine was administered 15 min before NE in the group “NE+Butox + Meth” or 45 min before Meth in the group “Butox + Meth”. PC12 cells were stained at 72 h after Meth. (**A**) Graph reporting TB staining; (**B**) graph reporting H&E staining; (**C**) graph reporting FJB staining. Data are given as the mean + SEM of nine independent counts for TB; six independent counts for H&E and FJB. Inferential statistics was carried out with ANOVA with Scheffè’s post hoc analysis. (DF = 15) * *p* < 0.05 compared with controls. ** *p* ≤ 0.05 compared with Meth.

**Figure 5:**
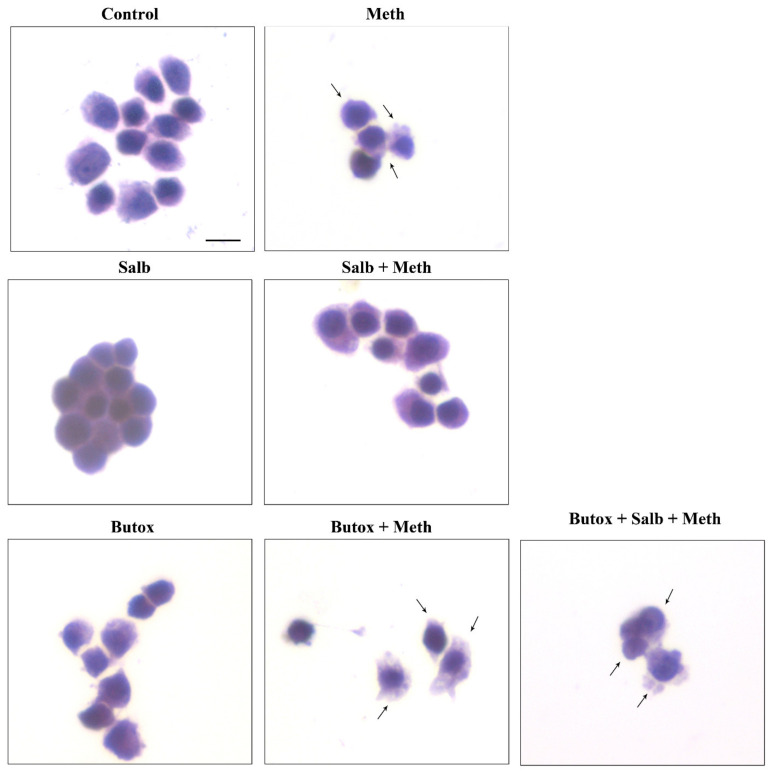
The β2-AR agonist (salbutamol) protects against Meth toxicity, while the β2-AR antagonist (butoxamine) impedes such a protection (H&E staining). Representative pictures show that salbutamol (5 μM) protects against Meth-induced toxicity. This β2-AR agonist is no longer protecting when the selective β2-AR antagonist (butoxamine) is administered. Butoxamine alone (10 μM) does not alter cell viability. Arrows indicate pale eosinophilic cells. Scale bar = 8 μM.

**Figure 6:**
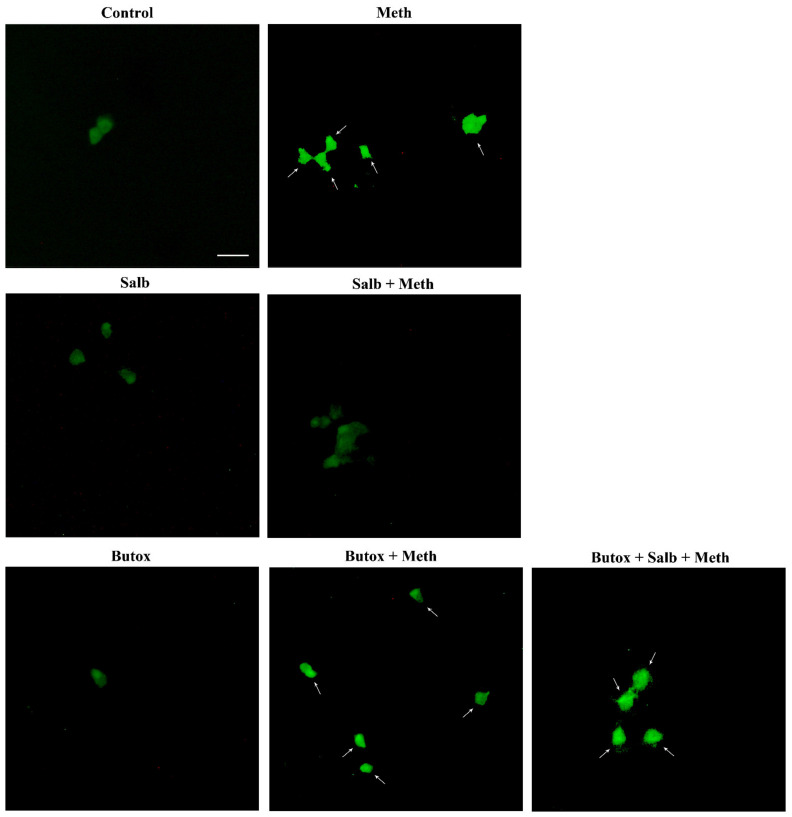
The β2-AR agonist (salbutamol) protects against Meth toxicity, while the β2-AR antagonist (butoxamine) impedes such a protection (FJB staining). Representative pictures show that salbutamol (5 μM) protects against Meth-induced toxicity. In fact, intensely fluorescent Meth-treated PC12 cells turn out into dark cells when salbutamol is pre-administered. This β2-AR agonist is no longer protecting when the selective β2-AR antagonist (butoxamine) is administered. In fact, cells administered salbutamol + butoxamine + Meth revert into intensely yellow fluorescence. Arrows indicate FJB intensely positive cells. Scale bar = 20 μM.

Once the role of β-AR was documented, the study specifically focused on which subtype of β-AR was responsible for protection. We observed that the selective β2-AR antagonist butoxamine (10 μM) completely occludes the protective effects of NE on Meth-induced toxicity (graphs of Figure 4 and representative pictures of Figure 5 and Figure 6). Pre-administration of butoxamine alone does not modify the toxicity induced by Meth. This is consistent with the pilot study showing that in the present experimental conditions, such a dose of Meth produces full toxicity. These data were confirmed by pre-administering the selective β2-AR agonist salbutamol (5 nM), which in fact fully protects against Meth toxicity, similarly to NE (Figure 4, Figure 5 and Figure 6). Altogether, these data indicate that β2-ARs are the sub-type of ARs, which are sufficient to replicate NE-induced protection against Meth-induced toxicity.

### 2.4. Is There a Protective Role for Intracellular NE?

Data obtained so far demonstrate that NE directly counteracts Meth toxicity. In addition, these data show that this effect fully relies on the activation of β2-AR. Nonetheless, when considering these ad interim conclusions, it remains unquestioned the issue of extracellular versus intracellular NE. In fact, when adding NE or specific ligands for ARs to the cell culture, a potential penetration of NE within the cell could contribute to neuroprotection or it may counteract a bigger or even an opposite effect. Therefore, we administered the selective NET blocker, DMI, alone or in combination with NE, to measure its effects in Meth-induced toxicity. 

**Figure 7:**
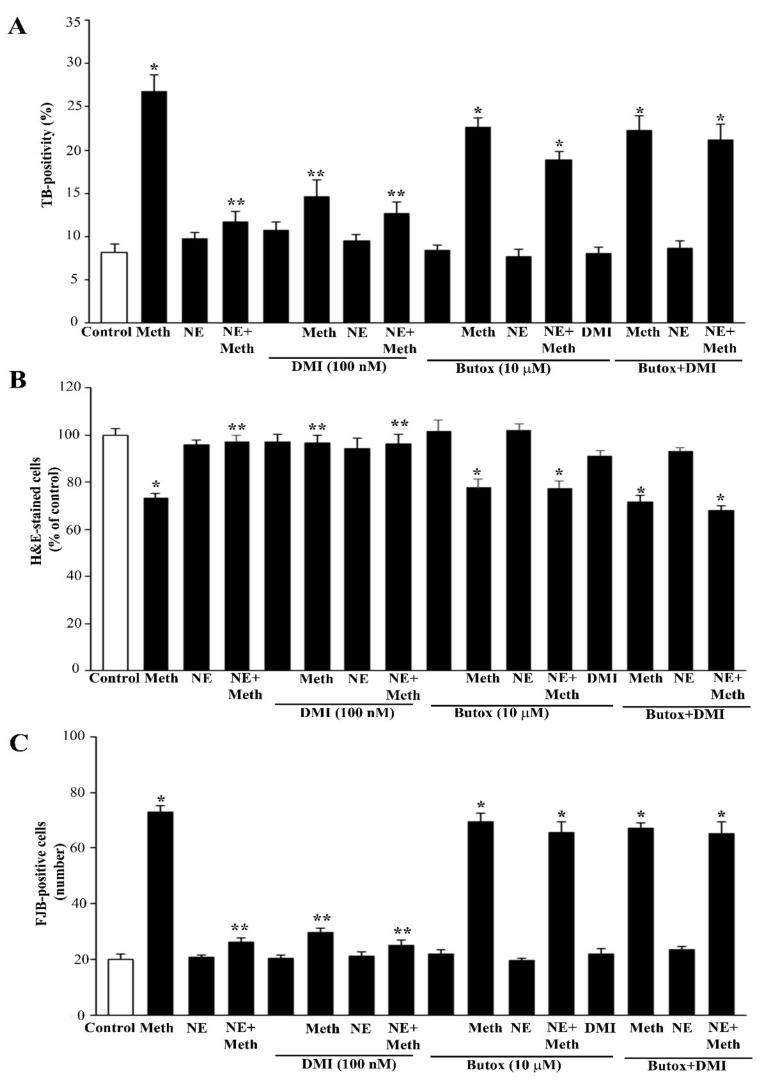
Protection of the NET blocker DMI on Meth toxicity through β2-AR is reverted by butoxamine. When the blocker of the NE transporter (NET) desmethylimipramine (DMI) was administered at the dose of 100 nM to PC12 cells, no effect could be detected. However, DMI 100 nM markedly protects against toxicity induced by Meth 50 μM to an amount that was comparable to NE 5 nM. Such an effect was lost when the β2-AR antagonist butoxamine 10 μM was pre-administered. This experiment was carried out to assess the contribution of extracellular vs. intracellular NE to protect against Meth toxicity. One might argue that an NET blocker reduces the amount of Meth that enters the cells. However, the passive diffusion of Meth and the entry through the dopamine transporter (DAT) are expected to overcome such a caveat. Most significantly, when the protection was due to a block in the entry of Meth, this could not be reversed by the β2-AR antagonist butoxamine. These data indicate that NE needs to act externally to the cell by activating β2-AR in order to protect against Meth toxicity. When co-administered with Meth, NE (5 nm) was applied 30 min before Meth; DMI (100 nM) or butoxamine (10 μM) were administered 45 min before Meth in the combined groups. (**A**) Graph reporting TB staining; (**B**) graph reporting H&E staining; (**C**) graph reporting FJB staining. Data are given as the mean+SEM of nine independent counts for TB; six independent counts for H&E and FJB. Inferential statistics was carried out with ANOVA with Scheffè’s post hoc analysis. (DF = 15). * *p* < 0.05 compared with controls. ** *p* ≤ 0.05 compared with Meth.

**Figure 8:**
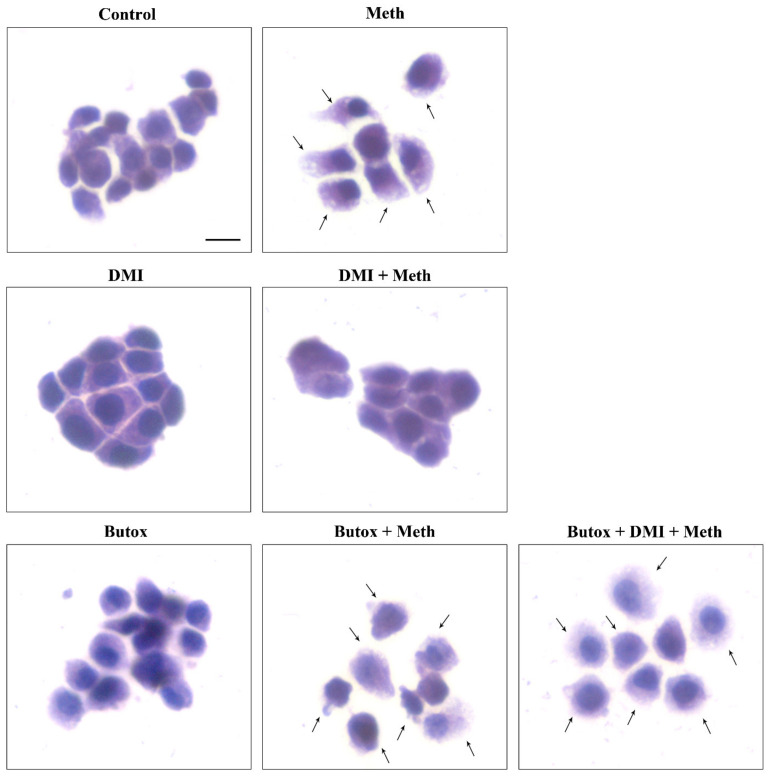
The NET blocker DMI protects against Meth toxicity through β2-AR (H&E). Representative pictures show that the NET inhibitor DMI (100 nM) protects against Meth (50 μM)-induced toxicity. This NET blocker is no longer protecting when the selective β2-AR antagonist (butoxamine) is administered. Butoxamine (10 μM) does not alter cell viability. Arrows indicate pale eosinophilic cells. Scale bar = 8 μM.

**Figure 9:**
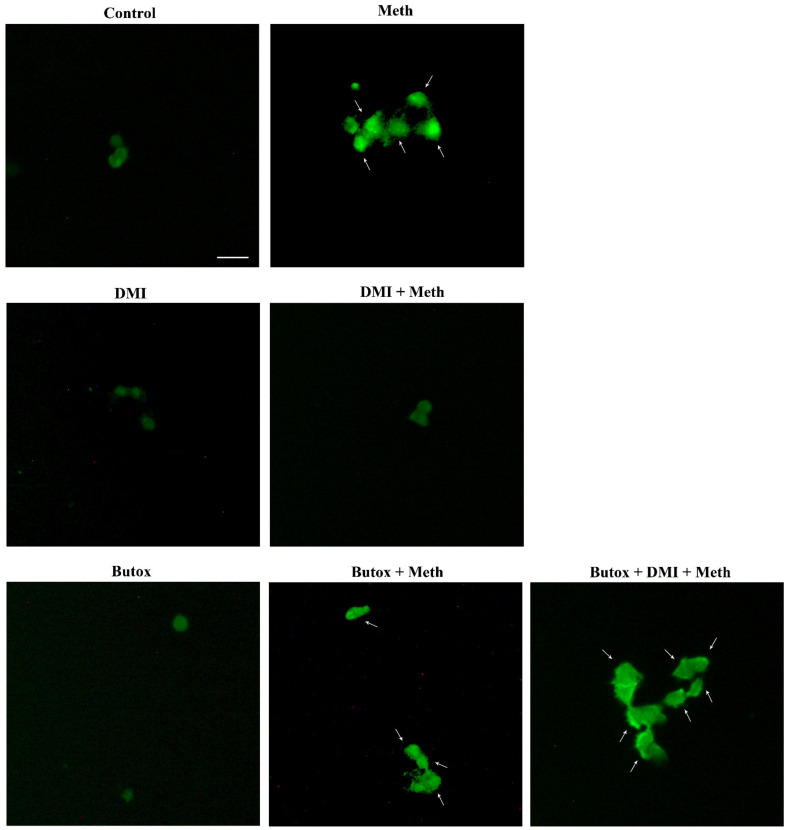
The NET blocker DMI protects against Meth toxicity through β2-AR (FJB). DMI (100 nM) administration obscures intense histofluorescence by FJB staining induced during Meth toxicity. Histofluorescence is brought back in intensity by the β2-AR antagonist butoxamine (10 μM), which re-instates Meth (50 μM)-induced toxicity. Arrows indicate FJB intensely positive cells. Scale bar = 20 μM.

DMI did not alter NE-induced protection against Meth toxicity (Figure 7, Figure 8 and Figure 9). Nonetheless, when DMI (100 nM) was co-administered with Meth, a full protection against Meth toxicity was observed, which is likely to depend on the increase in extracellular NE provided by this NET inhibitor (Figure 7, Figure 8 and Figure 9). 

It needs to be considered that both NE and DMI administered alone produce a full protection against Meth toxicity, which is in line with the rise of extracellular NE produced by DMI in PC12 cell lines, which exceeds 10 nM. Both protections were abolished by pre-administering the β2-AR antagonist butoxamine (Figure 7). These findings confirm the specific protective effects of β2-ARs.

In line with this, the protective effects of NE and DMI did not cumulate, which indicates a similar mechanism of protection, which is saturated either by NE (5 nM) or DMI (100 nM) (Figure 7). Consistently, the selective β2-AR antagonist butoxamine fully suppresses the protective effects of both DMI and NE on Meth-induced toxicity. These findings are consistent along various procedures (TB, H&E, FJB, Figure 7A–C and Figure 9), which indicates that NE-induced protection is fully based on the activation of plasma membrane β2-ARs.

**Figure 10:**
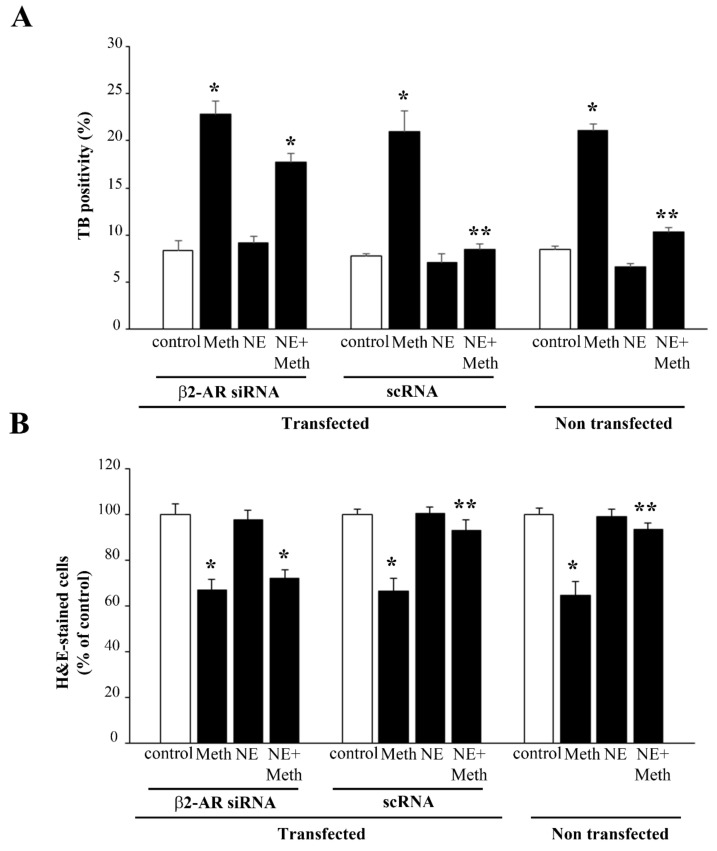

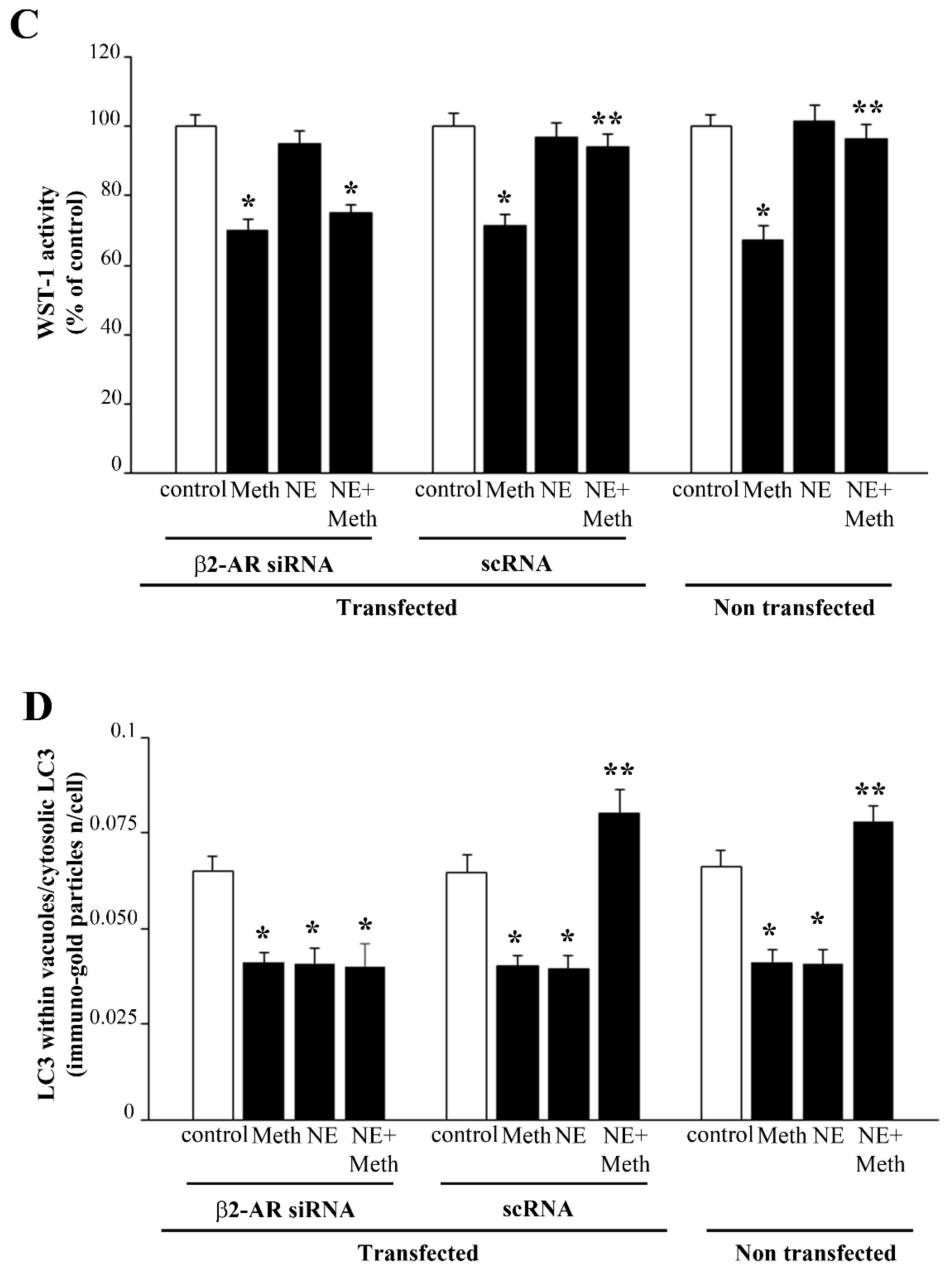
Transfection with β2-AR siRNA suppresses the protective effects of NE against Meth. Cell viability in naive and β2-AR siRNA- and scRNA-transfected cells, as evaluated by (**A**) TB, (**B**) H&E, and (**C**) WST-1 assay. (**D**) Dissipation of LC3 particles from vacuoles to the cytosol in β2-AR silenced cells. (DF = 11) * *p* < 0.05 compared with control; ** *p* < 0.05 compared with Meth.

**Figure 11:**
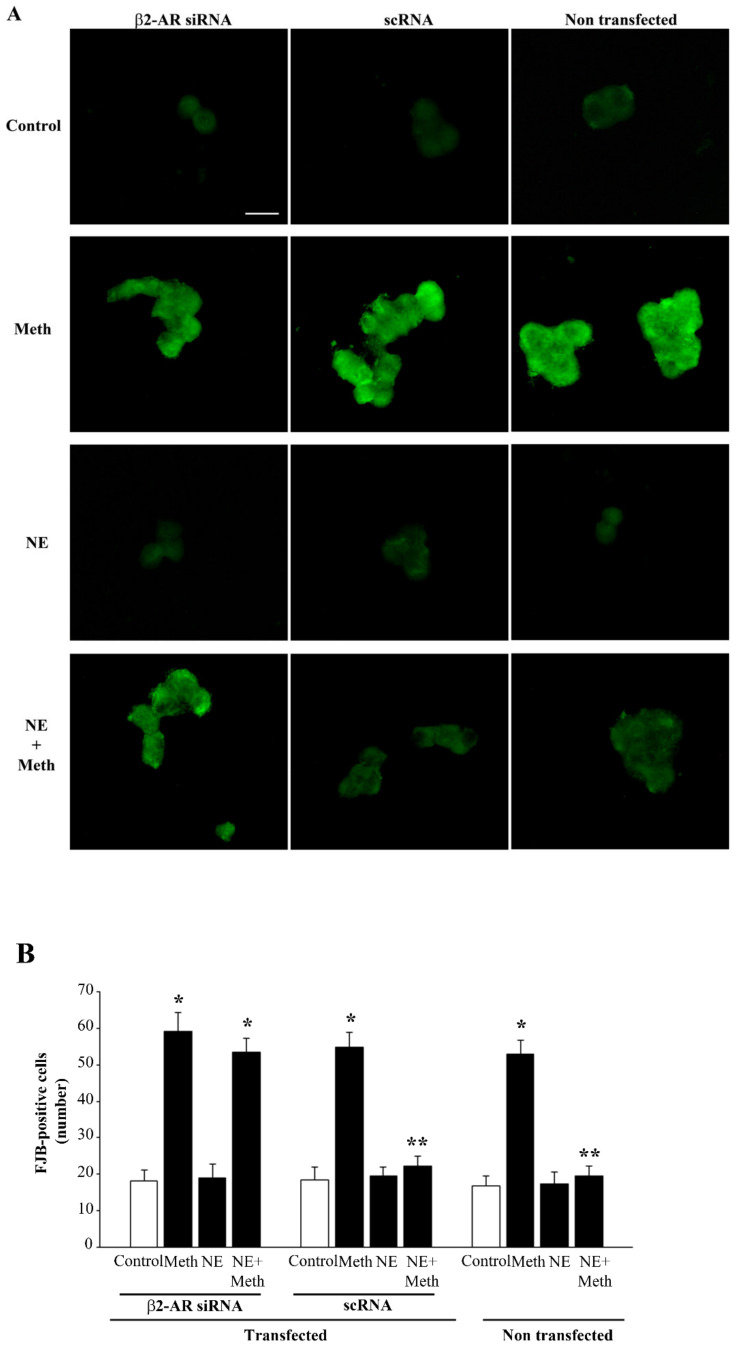
Meth-induced FJB fluorescence is not reduced by NE after transfection with β2-AR siRNA. (**A**) Representative pictures and FJB fluorescent cells showing the effects of Meth and NE in naïve and β2-AR siRNA- and scRNA-transfected cells. (**B**) The graph reports the number of FJB fluorescent cells in the different experimental groups. (DF = 11) * *p* < 0.05 compared with control; ** *p* < 0.05 compared with Meth. Scale bar = 13.3 μM.

As a further proof, these findings were challenged by transfecting cells by silencing RNA (siRNA) for β2-ARs. The efficacy of transfection was very high (over 75%) as shown by Supplementary Figure S10 (immunoblotting) and S11 (immunogold). Silencing β2-ARs suppresses the neuroprotective effects of NE against Meth toxicity (Figure 10 and Figure 11). Consistently, even DMI-induced protection was lost in β2-ARs silenced cells (cell death: 25.2% ± 2.7% in Meth-treated WT cells, 25.0% ± 3.1% in β2-ARs siRNA NE + Meth-treated cells).

### 2.5. NE Counteracts Meth-Induced Dissipation of LC3 from Autophagy Vacuoles

**Figure 12:**
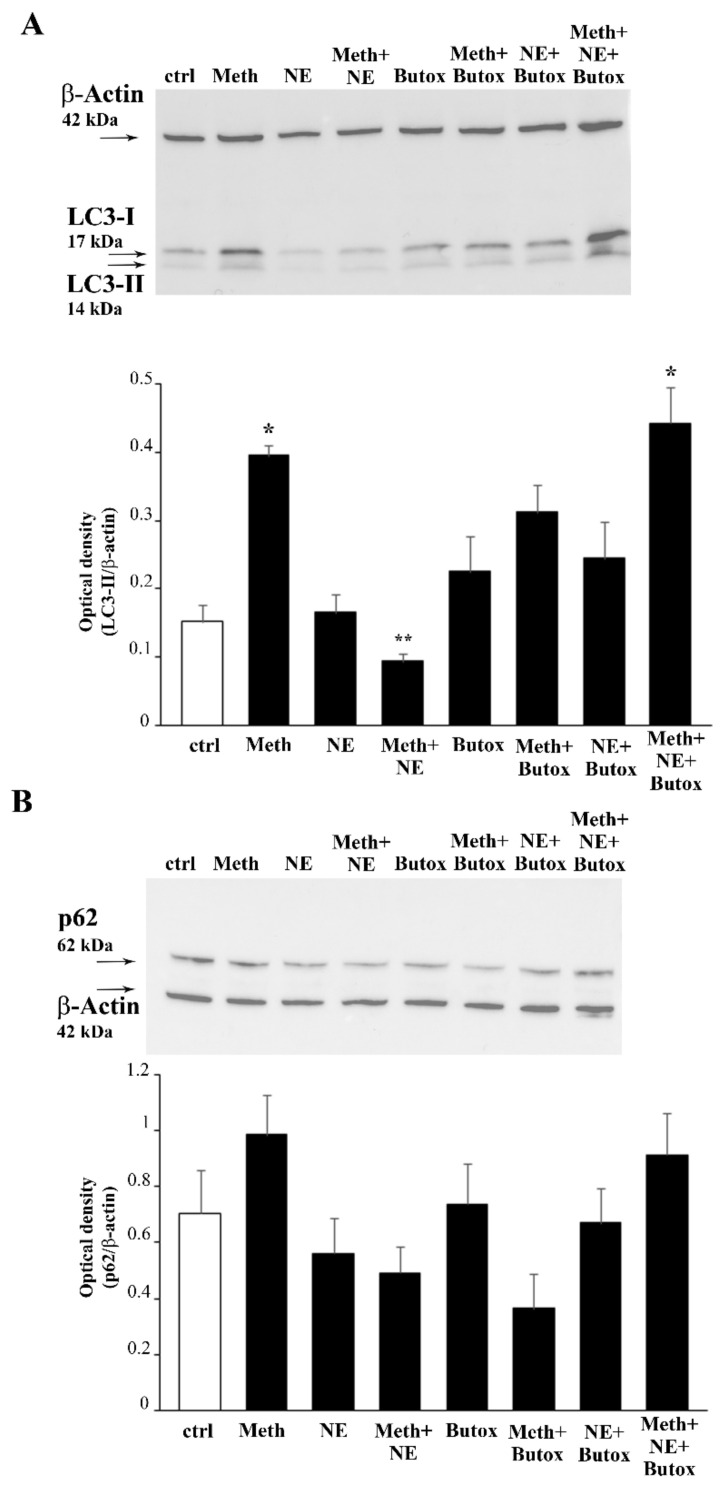
Opposite effects of NE and the β2-AR antagonist butoxamine on the LC3-II and p62 accumulation induced by Meth. Western blot for the autophagy markers LC3-I and LC3-II (**A**) and p62 (**B**) are reported along with the related optical densities. (DF = 7). * *p* < 0.05 compared with controls; ** *p* < 0.05 compared with Meth. Ctrl = control.

**Figure 13:**
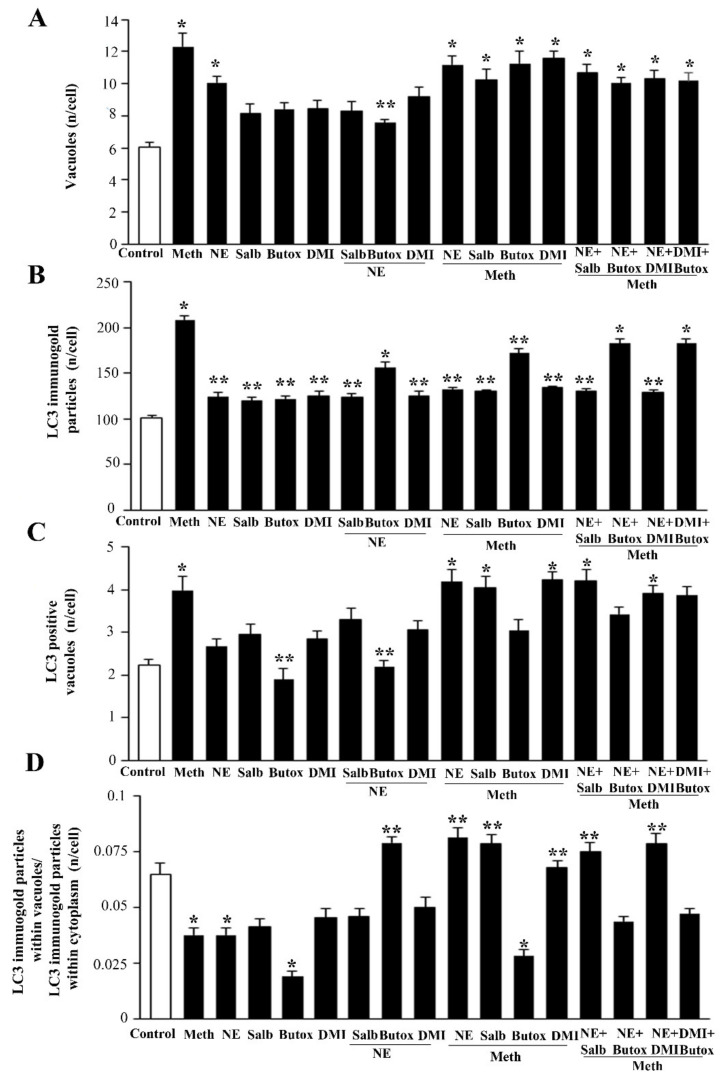
Effects of NE, Meth, β2-AR ligands, and DMI on autophagy vacuoles and LC3 compartmentalization. The graphs show (**A**) the amount of authentic autophagy vacuoles (defined as double/multiple membrane vacuoles containing LC3 molecules); (**B**) the amount of whole cell LC3 immunogold particles; (**C**) LC3 positive vacuoles; (**D**) the ratio between LC3 within vacuoles and whole cell LC3. Measurements are obtained by ultrastructural morphometry where the quantitative measurement of vacuoles and stoichiometrically identified LC3 particles by immunogold are counted. The graphs show that Meth increases the amount of autophagy-like vacuoles and autophagy vacuoles along with whole cells LC3 particles compared with control. Nonetheless, the ratio of LC3 within vacuoles and LC3 in the whole cell is strongly diminished. This indicates a de-polarization of LC3 from vacuoles to the cytosol. Such an effect is reversed by NE, which by itself does not increase polarization compared with controls. Salbutamol replicates the data obtained with NE, as it does DMI, while butoxamine occludes these effects. Numbers are given as the mean+SEM of autophagy-like vacuoles/autophagy vacuoles/cytosolic LC3 particles/vacuolar LC3 particles per cell, which were counted in 50 cells per group. Inferential statistics was carried out with ANOVA with Scheffè’s post hoc analysis. (DF = 16). * *p* < 0.05 compared with controls. ** *p* ≤ 0.05 compared with Meth.

**Figure 14:**
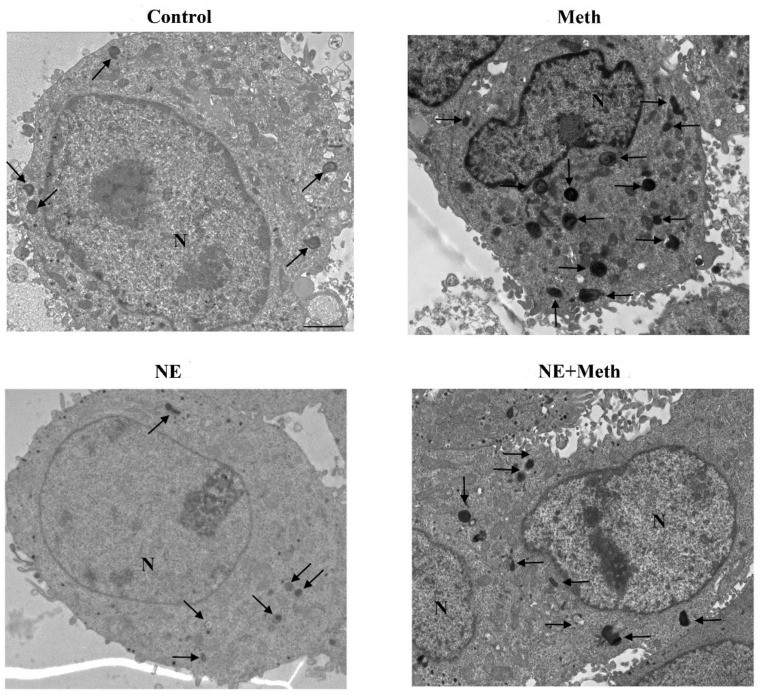
Representative TEM micrograph following NE and Meth of autophagy-like (low magnification). Representative TEM micrographs at low magnification of Control, Meth (50 μM), NE (5 nM), or NE (5 nM) + Meth (50 μM)-treated cells. Images show autophagy-like vacuoles. These correspond to vacuolar inclusions with double or multiple limiting membranes possessing the same electron density of cytosol sometimes interrupted by electron dense remnants. Autophagy-like vacuoles indicated by an arrow are increased in the cytosol by Meth, far exceeding the amount observed in Control or NE-treated cells. PC12 cells were fixed for TEM analysis 72 h after Meth administration. In the combined treatment, NE was administered 30 min before with Meth. Scale bar = 1 μM. N = nucleus.

**Figure 15:**
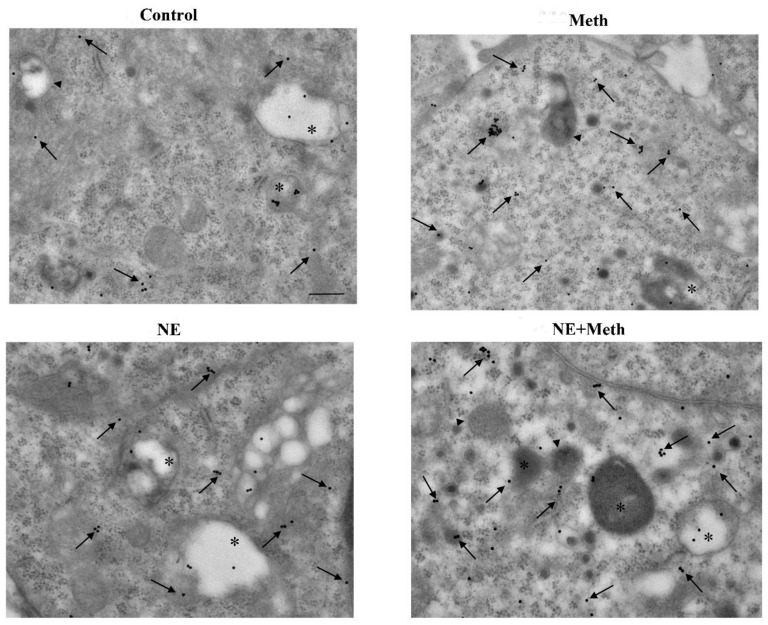
Representative TEM micrograph of LC3 compartmentalization following NE and Meth (high magnification). Representative TEM micrographs at high magnification of Control, Meth (50 μM), NE (5 nM), or NE (5 nM) + Meth (50 μM)-treated cells. Images show autophagy-like vacuoles (arrowhead); autophagy vacuoles (asterisk for LC3); and cytosolic LC3 particles (arrow pointing 20 nM immunogold particles). Cells were fixed for TEM analysis 72 h after Meth administration. In the combined treatment, NE was administered 30 min before with Meth. Scale bar = 200 nM.

The administration of Meth 50 μM inhibits the autophagy flux, as indicated by the increase in the levels of the autophagy markers LC3-II and p62 (Figure 12). This is concomitant with an increase in autophagy vacuoles, LC3 particles, and LC3-positive vacuoles (Figure 13, Figure 14 and Figure 15). This is due to an inhibition of autophagy progression. 

This significant increase in whole cell stoichiometrically counted LC3 immunogold particles (Figure 13B) and LC3-positive autophagy vacuoles (Figure 13C) following Meth does not occur in parallel. In fact, a strong decrease in the ratio of LC3 within vacuoles and LC3 within the cytosol is counted (Figure 13D and representative Figure 15). Thus, Meth dissipates LC3 particles from autophagy vacuoles toward the whole cell (Figure 13). Remarkably, NE fully reverts this effect (Figure 12A and Figure 13D).

**Figure 16:**
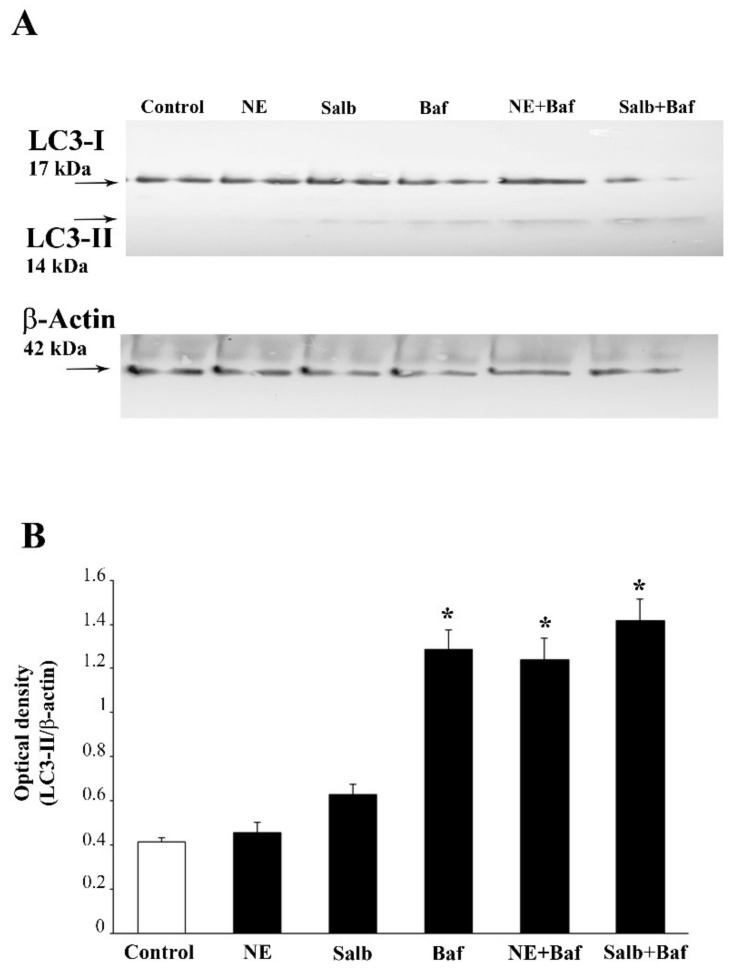
NE and the β2-AR agonist salbutamol do not counteract the inhibition of the autophagy flux produced by bafilomycin. Western blot for LC3-I and LC3-II (**A**) and related optical density (**B**) are reported. (DF = 5). * *p* < 0.05 compared with controls.

In fact, the administration of a fully protective dose of NE re-establishes the ratio between vacuoles and cytosol for LC3 (Figure 13D and representative Figure 15). In detail, NE induces a polarization of LC3 within vacuoles, which surpasses that counted in control cells. This recruitment of LC3 within vacuoles is contingency-dependent, since it does not occur when it is not needed. In fact, when administered alone, NE does not produce a compartmentalization, and it only slightly increases unstained autophagy vacuoles (Figure 13A and representative Figure 14); the polarization of LC3 only occurs in the presence of a stressful insult. In particular, following exposure to NE alone, the number of autophagy-like vacuoles increases compared with control cells (Figure 13B and representative Figure 14). However, NE in the absence of Meth does not induce a parallel increase in total LC3 particles and LC3-positive vacuoles per cells (Figure 13B and representative Figure 14). This re-allocation of LC3 within autophagy vacuoles produced by NE in Meth-treated cells is concomitant with an NE-induced burst in the autophagy flux, despite NE alone not being effective in speeding autophagy flux in baseline condition (Figure 16). It is likely that in baseline conditions, the ratio of LC3 compartmentalization is just fine to grant an adequate effect, and adding NE in baseline conditions does not produce any additional effect to such a steady condition. 

### 2.6. β2-AR Activity Counteracts Meth-Induced LC3 Dissipation 

After showing the specificity for β2-AR to counteract Meth-induced toxicity (see Section 2.3), and following the demonstration of the fine ultrastructural mechanisms responsible for NE-induced protection against Meth toxicity, we questioned whether the sub-cellular findings detected at ultrastructural morphometry were confirmed following the stimulation of β2-ARs. 

**Figure 17:**
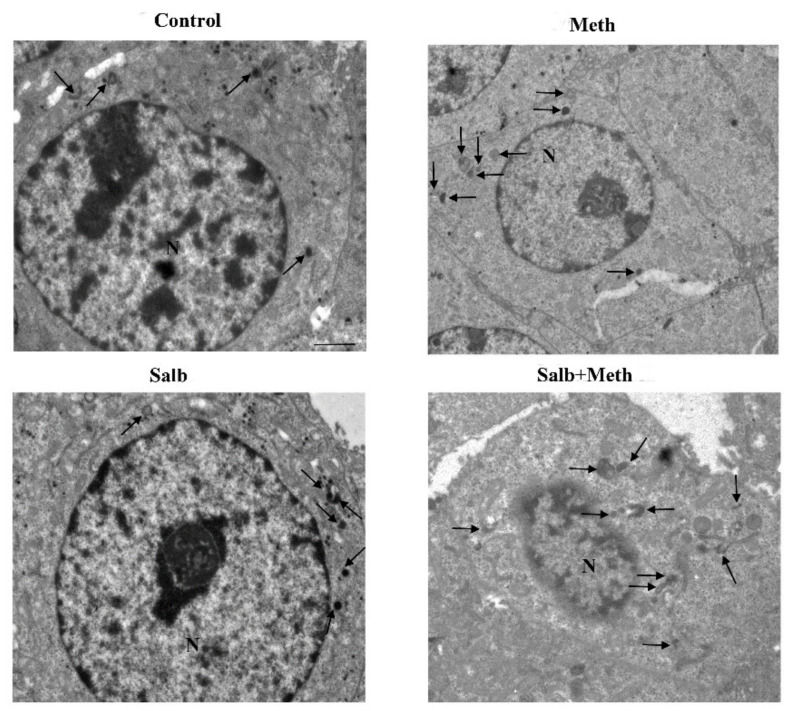
Representative TEM micrograph of autophagy-like vacuoles following salbutamol and Meth (low magnification). TEM micrographs at low magnification of Control, Meth (50 μM), salbutamol (5 nM), or salbutamol (5 nM) + Meth (50 μM)-treated cells. Images show autophagy-like vacuoles (arrow). Cells were fixed at 72 h after Meth administration. In the combined treatment, salbutamol was administered 30 min before with Meth. Scale bar = 1 μM. N = nucleus.

**Figure 18:**
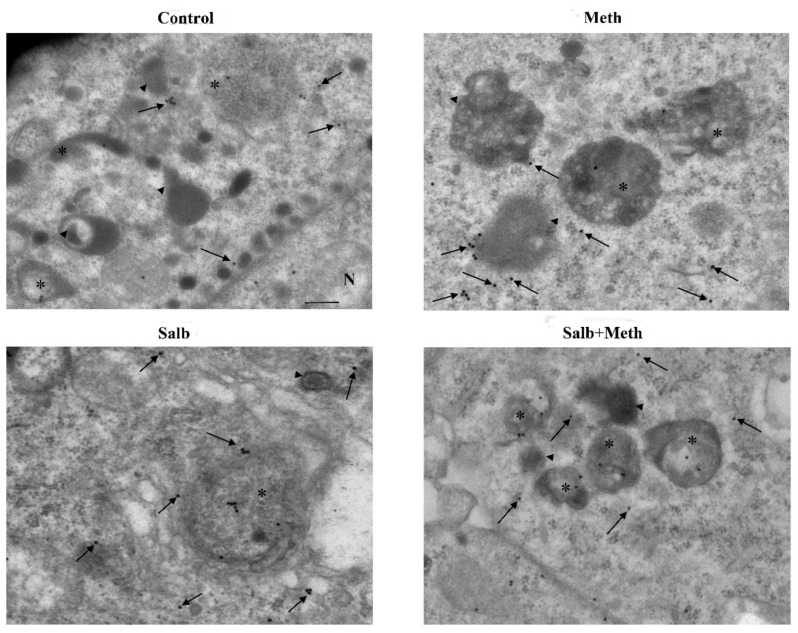
Representative TEM micrograph of LC3 compartmentalization following salbutamol and Meth (high magnification). Representative TEM micrographs at high magnification of Control, Meth (50 μM), salbutamol (5 nM), or salbutamol (5 nM) + Meth (50 μM)-treated cells. Images show autophagy-like vacuoles (arrowhead); autophagy vacuoles (asterisk for LC3); cytosolic LC3 particles (arrow pointing 20 nM immunogold particles). Cells were fixed for TEM analysis 72 h after Meth administration. In the combined treatment, salbutamol was administered 30 min before with Meth. Scale bar = 200 nM.

Administration of the selective β2-AR agonist salbutamol mimics the re-allocation of LC3 compartmentalization operated by NE (Figure 13D and representative Figure 17 and Figure 18). Contrary wise, the β2-AR antagonist butoxamine prevents NE from counteracting Meth-induced LC3 dissipation (Figure 13D). Thus, the block of β2-ARs reverts the effects of NE on LC3 compartmentalization within autophagy vacuoles.

Butoxamine alone induces a slight, non-significant increase of total LC3 particles per cell, which occurs in parallel with a decrease of the ratio between vacuolar/cytosolic LC3 (Figure 13D).

### 2.7. The Effects of DMI on Meth-Induced LC3 Dissipation 

**Figure 19:**
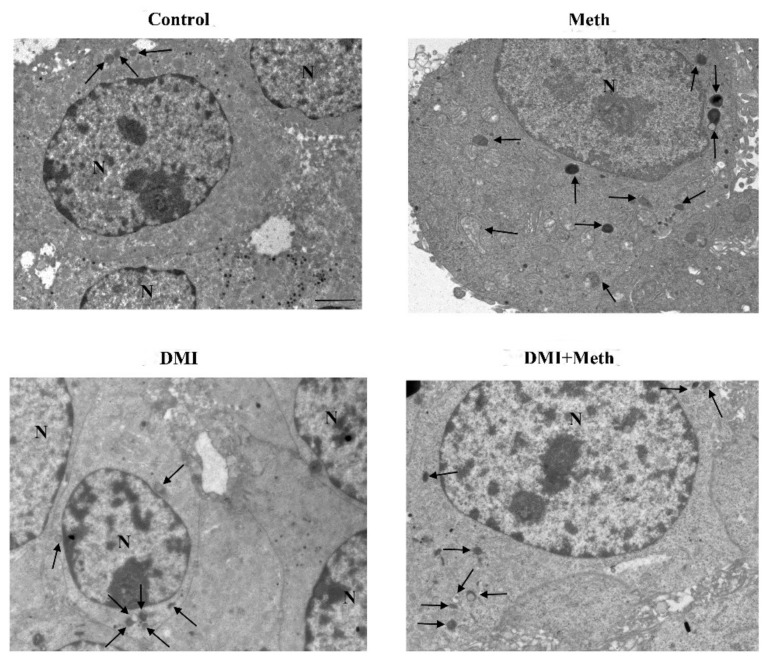
Representative TEM micrograph of autophagy-like vacuoles following DMI and Meth (low magnification). Representative micrographs of Control, Meth (50 μM), the selective NET blocker DMI (100 nM), or DMI (100 nM) + Meth (50 μM)-treated cells. Cells were fixed at 72 h after Meth administration. In the combined treatment, DMI was administered 45 min before Meth. Autophagy-like vacuoles are shown (arrow). Scale bar = 1 μM. N = nucleus.

**Figure 20:**
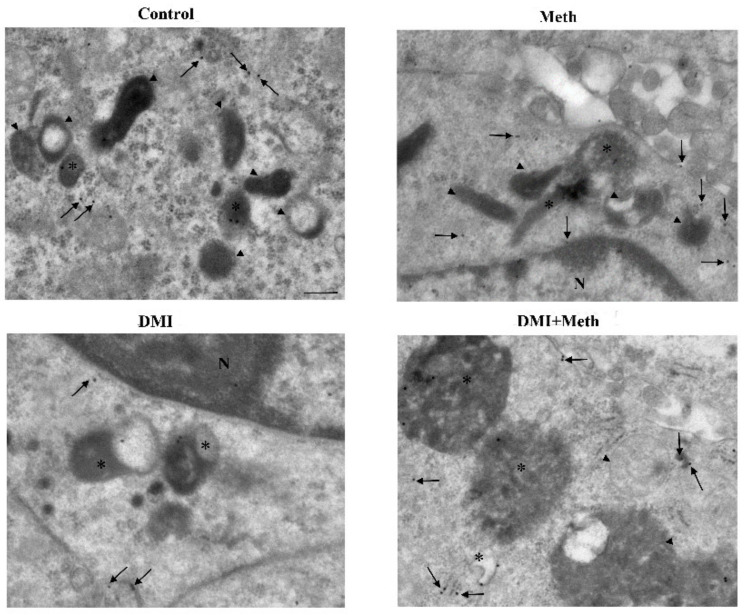
Representative TEM micrograph of LC3 compartmentalization following DMI and Meth (high magnification). Representative TEM micrographs at high magnification of Control, Meth (50 μM), the selective NET blocker DMI (100 nM), or DMI (100 nM) + Meth (50 μM)-treated cells. Images show autophagy-like vacuoles (arrowhead); autophagy vacuoles (asterisk for LC3); cytosolic LC3 particles (arrow pointing 20 nM immunogold particles). Cells were fixed for TEM analysis 72 h after Meth administration. In the combined treatment, DMI was administered 45 min before Meth. Scale bar = 200 nM.

When DMI was administered to Meth-treated cells, LC3 dissipation was prevented as measured following the administration of either NE or salbutamol to Meth-treated cells (Figure 13D, and representative Figure 19 and Figure 20). As expected, such an effect was occluded by the β2-AR antagonist butoxamine (Figure 13D).

## 3. Discussion

In the present study, evidence is provided that NE fully protects against Meth-induced cell death in PC12 cells. Such a protection is evident by combining various methods to detect cell viability/cell toxicity. It is remarkable that the FJB technique confirms results obtained with classic approaches to detect cell damage. This represents a methodological novelty, since FJB was never used in cell lines to detect Meth toxicity; thus, the present results also provide the validation of a novel experimental approach to detect Meth toxicity in vitro. 

Protection induced by NE is evident at the dose of 5 nM and 50 nM, in which the 5 nM dose is fully protective against Meth toxicity, even for a 2 mM dose of Meth. The protective effects of Meth can be replicated by administering the non-selective β-agonist, isoproterenol, while they are fully reversed by administering the non-selective β-antagonist propranolol. The protection induced by NE through β-AR stimulation is reproduced by activating the β2-AR subtype by administering the selective agonist salbutamol, which mimics the protection induced by either NE or isoproterenol. The specificity of β2-ARs activation to protect against Meth toxicity is confirmed by a loss of protection when NE is co-administered with the selective β2-AR antagonist butoxamine, which in turn replicates the effects produced by the non-selective β-antagonist propranolol. The final validation of the protective role induced by β2-ARs stimulation was demonstrated by silencing β2-AR, which prevents the protective effects of NE. These protective effects of NE depend on the stimulation of β2-AR placed on the plasma membrane, since an inhibition of NE uptake by the NET blocker DMI does not reduce NE-induced protection, despite preventing NE to enter into the cells. It is remarkable that the blockade of NET induces per se a protective effect, since apart from blocking Meth toxicity, it reduces the slight amount of cell death, which occurs spontaneously in baseline conditions in PC12 cells. One might argue that blocking NET prevents Meth from exerting its effects within PC12 cells, since NET represents one of the hooking sites for Meth to enter the target cells. However, most of the entry occurs by the simple diffusion of Meth [37]. Moreover, PC12 cells express a high amount of DAT [38,39] and serotonine transporter (SERT) [40], which are not blocked by DMI (a selective NET inhibitor), which leaves a wide gateway for Meth to enter the cells even in the presence of a NET blockade [41,42]. At any rate, the loss of protection produced by butoxamine in DMI + Meth-treated cells rules out a bias due to a block of Meth entry by DMI. In fact, if Meth was prevented from entering the cell, butoxamine could not re-instate Meth toxicity. Again, the occurrence of a protection against baseline ongoing slight toxicity in PC12 cells by DMI witnesses for the lack of an interference of DMI with Meth availability for the cells. In line with this, also, the protective effects of DMI were abolished following β2-AR silencing. 

The kind of receptors, which were probed in the present study, take into account the variety already described in these cells.

In fact, PC12 cells normally express α1- (specifically, the subtypes α1A and α1D), β1- and β2-ARs, as well as NET [43] and DAT [38]. This is consistent with data obtained in the present study. The full replication of NE protection against Meth toxicity measured here through the activation of both non-selective β-AR and selective β2-AR agonists fully reciprocates the effects induced by a non-selective β-AR antagonist and a selective β2-AR antagonist. This made it redundant to challenge Meth toxicity with other β-AR ligands such as specific β1-ARs, which was not investigated in the present study. At this stage of the investigation, the issue of which AR mediates the protection by NE seems to be solved. Nonetheless, considering other receptors being expressed by PC12 cells, we were curious to probe the role of α1-AR. In fact, α1-AR are implicated in paradoxical deleterious effects, which may be induced by NE in vivo within specific contingencies. This regards α1-AR-mediated neurotoxicity and neurodegeneration, which we contributed to describing [44,45]. Therefore, one might argue that despite NE protecting against Meth toxicity and β2-ARs fully substituting for the effects produced by NE, there might be still a detrimental effect mediated by α1-AR, which is working during NE stimulation. These effects could be obscured by the overwhelming beneficial activity exerted by the stimulation of β2-ARs. 

To explore such a potential slight discrepancy, we challenged the experimental settings with the selective α1-AR agonist phenylephrine. Additionally, to disclose a potential contraction of the full protective potential of NE by a concomitant opposite activity generated by the activation of α1-Ars, we co-administered the α1-AR antagonist prazosin. In each experimental condition, any noticeable alteration of neither Meth toxicity nor NE protection against Meth toxicity was observed. These latter findings deserve some room for a brief comment since, the lack of a slight detrimental influence in the beneficial scenario provided by NE may be attributed to various facets. At first, the detrimental role of α1-AR has been shown in vivo, where it is believed to occur via a trans-synaptic loop, which does not directly produces toxicity on target cells [46].

Again, a potential role of β3-ARs requires further investigations concerning the protective efficacy of NE, since these ARs remain much less investigated compared with β1- and β2-ARs, and β receptors ligands may recruit β3-ARs to a certain amount. This is discussed elegantly by Baker (2010) [47] in *The British Journal of Pharmacology*, but it is further reported by recent papers [48]. 

Nonetheless, the results provided here through the specific silencing of β2-ARs tend to rule out any potential role for β3-ARs.

In considering the potential dual effects in vivo produced by the stimulation of a variety of ARs in modulating Meth toxicity, it should be kept in mind that in vivo, in different animal species, the loss of NE leads to an enhancement of Meth toxicity [2,3,4,6,49,50].

**Figure 21:**
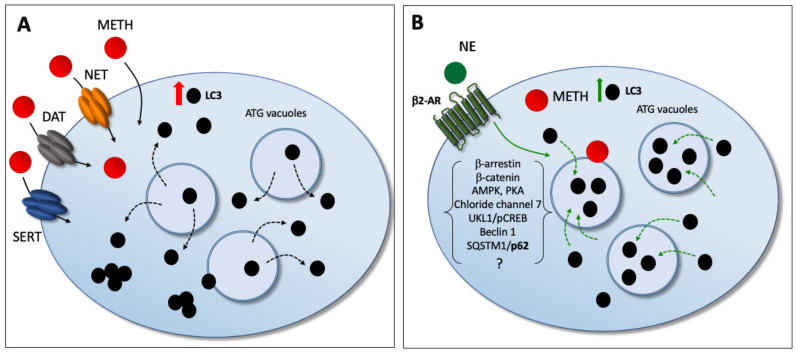
The cartoon summarizes the protective effects of NE against Meth-induced toxicity. (**A**) In detail, Meth moves out LC3 molecules from autophagy vacuoles toward the cytosol, thus de-potentiating the autophagy machinery. (**B**) NE, by acting on β2-AR, counteracts Meth-induced toxicity while re-polarizing LC3 toward autophagy vacuoles.

Nonetheless, an insight in their mechanisms is lacking at the subcellular level. Remarkably, the direct effect of NE in preventing Meth-induced toxicity is associated here with a specific compartmentalization of LC3 within autophagy vacuoles. In fact, NE exposure counteracts the dissipation of LC3 from autophagy vacuoles toward the cytosol, which is induced by Meth. In detail, Meth induces the overexpression of LC3 within PC12 cells. However, LC3 moves out from autophagy vacuoles to fill non-compartmentalized cytosolic regions (as reported in the explanatory cartoon of Figure 21). The stoichiometric measurement of LC3 by immunogold was not modified by NE administration. However, NE moves back LC3 within vacuoles, which is expected to restore competence to the autophagy machinery. These data mimic what was recently demonstrated for the protective effects of the powerful autophagy activator rapamycin in Meth-intoxicated PC12 cells [30]. 

Similar findings were obtained by administering salbutamol, which confirms the β2-ARs dependency of the whole effect induced by NE; in line with this, butoxamine pre-administration prevents LC3 polarization within vacuoles, which was induced by NE in PC12 cells.

Similar results were obtained in β2-ARs silenced cells.

It remains a matter for future studies to further explore the transduction pathways bridging the activation of β2-ARs to such a modulation in the trafficking and compartmentalization of LC3. A link between β2-ARs activation and autophagy occurs in fibroblasts [51], cardiomyocytes [52], hepatic cells [53], and gastric cells [54]. In particular, Aránguiz-Urroz et al. (2010) [51] showed that NE, isoproterenol, and salbutamol all increase the autophagy flux in cultured adult rat cardiac fibroblasts, and these effects are blocked both by propanolol and a selective β2-AR antagonist (ICI-118,551), while drugs acting on β1-AR do not modify these effects. More recently, Farah et al. (2014) [53] showed that the β2-AR increases autophagy by elevating cAMP levels. The activation of β2-AR may stimulate autophagy also via an increased phosphorylation of AMPK [55]. Very recently, β2-AR activation was shown to activate autophagy flux along with increased double-membrane vesicles, punctuating GFP-RFP-LC3 distribution in the cells [54]. This was also related to the activation of cAMP response element binding (CREB) protein, leading to autophagy activation through the adenosine 5′ monophosphate activated protein kinase ULK1 (AMPK ULK1) pathway [54,56]. The activation of β2-ARs increases β-arrestin, β-catenin, Beclin1, Chloride channel 7, and SQSTM1/p62 [56,57,58,59,60,61,62]. All these complex and interconnected biochemicals cascades should be studied in order to comprehend a potential role in the ultrastructural morphometry measured here.

A specific dissection of the effects of NE on autophagy pathways remains beyond the aims of this study, as well as the concomitant role of other ARs. Nevertheless, the present observation might be relevant to understand why a loss of brain NE fosters the onset of disorders induced by deranged autophagy activity [63]. This occurs in neurodegenerative disorders ranging from movement disorders to degenerative dementias. In fact, it is well known that NE depletion potentiates the degeneration of the DA nigrostriatal pathway [1,2,3,49,50,64,65,66]. It is remarkable that in these disorders, the loss of NE anticipates the onset of disease symptoms [67,68], suggesting that the loss of NE fosters degeneration [7,10,69]. In fact, in vivo, a loss of NE fibers dramatically potentiates Meth-induced neurotoxicity [2,3,50,70]. 

## 4. Materials and Methods

### 4.1. Cell Cultures and Experimental Design

The PC12 cell line was purchased from a cell bank (IRCCS San Martino Institute, Genova, Italy). Cells were grown in RPMI 1640 medium (Sigma-Aldrich, St. Louis, MO, USA), which was supplemented with heat-inactivated, 10% horse serum (HS, Sigma), 5% fetal bovine serum (FBS, Sigma), and penicillin (50 IU/mL)/streptomycin (50 mg/mL, Sigma), and they were kept under standard culture conditions in a humidified atmosphere containing 5% CO_2_ at 37 °C. Experiments were carried out during what it appeared to be the cell growth log-phase, when the cells had reached approximately 70% confluence [71,72]. Cells were seeded within plates to be further incubated at 37 °C in 5% CO_2_ for 24 h. This brought up the number of cells used for TEM experiments to a magnitude that allowed reaching an amount of cells to be further seeded in a number of roughly 10^6^ per each experimental group. Each experimental group was seeded in a culture dish to reach a final volume of 5 mL, including the supplemented medium. 

In a preliminary set of experiments, we assessed the effects of NE on Meth-induced toxicity by light microscopy, by using TB, H&E, and FJB staining. We also used a colorimetric cell viability assay as a complementary technique to the latter ones, namely the WST-1 cell viability assay. 

We first treated PC12 cells with different doses of Meth (Sigma, 5 μM, 50 μM, and 100 μM) for 72 h. This dose of Meth 50 μM induces roughly 25% cell loss, which corresponds to a plateau, since the 100 μM dose does not produce any further cell loss in the present experimental settings. Then, the effects of NE (Sigma) at different concentrations (0.5 nM, 5 nM, and 50 nM) were measured in PC12 cells treated with 50 μM Meth. Each dose of NE was administered 30 min before Meth. In this way, a short dose–response effect curve for NE modulation of Meth-induced toxicity was carried out. Then, in order to test the role of specific NE receptors, we tested cell viability following specific β- or α-AR agonist and antagonists, since PC12 have been shown to express α1- (specifically, the subtypes α1A and α1D), β1-, and β2-AR [43]. As a non-selective β-AR antagonist, we administered propranolol 50 μM, while as an agonist, isoproterenol 400 μM was administered. As a selective β2-AR antagonist, butoxamine 10 μM was administered, while the selective β2-AR agonist salbutamol was administered at the dose of 5 nM. To test the role of α-AR, we administered the α1-AR agonist phenylephrine 10 μM as well as the α1-AR antagonist prazosin 15 μM. All these compounds were tested alone or in combination with each other and/or with NE 5 nM, 30 min before Meth 50 μM, to test the modulation of toxicity. Finally, to assess the potential contribution of intra-cellular NE, we concomitantly administered desmethylimipramine (DMI, 100 nM, Sigma), a selective blocker of the membrane NE transporter (NET) (which is expressed in PC12; [43]), alone or in combination with the β2-AR antagonist butoxamine, 15 min before NE, and/or 45 min before Meth. The doses of various agonists and antagonists at NE receptors correspond to those reported to produce a full receptor occupancy as follows: for phenylephrine [43]; for prazosin [73]; for butoxamine [74]; for salbutamol [75]; for isoproterenol [76]; for propranolol [77,78]; for DMI [79,80] also according to drug bioactivity databases (see link in [81]). 

Once the modulation of Meth-induced toxicity was established at light microscopy, an early step of autophagy recruitment specifically altered by Meth was investigated. This was carried out both for NE and NE ligands, which modulate Meth-induced toxicity. The recruitment of the autophagy machinery was better detailed and quantified by using ultrastructural morphometry at transmission electron microscopy (TEM). 

The protective effects of NE on Meth intoxication were confirmed in a simple cell system, and they could be mostly attributed to the activation of β2-AR; the molecular mechanisms responsible for these effects were investigated in the main section of the study. This was carried out by ultrastructural morphological analysis of the effects of NE and β2-AR ligands on the casting of the LC3-filled autophagosome. In detail, we analyzed the compartmentalization of LC3 particles and their polarization toward autophagy vacuoles. All these experiments were carried out at 72 h following 50 μM Meth exposure. For this experimental section, we selected the 5 nM dose of NE, since this corresponds to the one that provides the plateau in protecting against Meth-induced toxicity. Butoxamine, salbutamol, and DMI were used at the concentrations already used for TB, H&E, FJB, and WST-1 analysis already described above.

### 4.2. Cell Cycle Analysis by Flow Cytometry 

The cell cycle was analyzed by DNA fluorescence flow cytometric profiles following the method described by Nicoletti et al. [82]. Briefly, cells detached from plates were washed two-fold in PBS and then resuspended in 1 mL of fluorochromic solution containing 0.05 mg/mL PI (propidium iodide), 0.1% sodium citrate, and 0.1% Triton X-100 and then placed at 4 °C in the dark overnight before the flow-cytometric analysis. The fluorescence of DNA of isolated nuclei (PI fluorescence) was analyzed by Cytoflexflow cytometer (Beckman Coulter, Cassina de’Pecchi, Milano, Italy).

### 4.3. Silencing RNA Transfection

Small silencing RNAs targeting β2ARs gene were obtained from Ambion (Life Technolgies, Carlsbad, CA, USA). The siRNA sequences used were as follows: sense 5′GCUGUGACUUCUUCACGAAtt3′; antisense 5′UUCGUGAAGAAGUCACAGCaa3′. As negative control (scramble RNA, scRNA), Silencer^®^ Select Negative Control #1 siRNA (catalogue #4390843, Life Technologies) was used.

PC12 cells were seeded onto poly-lysine covered multi-well plates as follows:

(i) For Western blot and TEM experiments, cells were seeded onto 6-well plates at a density of 1 × 10^6^ cells per well;
(ii) For light microscopy, cells were seeded onto 24-well plates at a density of 4 × 10^5^ cells per well;
(iii) For WST-1 assay, cells were seeded onto 96-well plates at a density of 1 × 10^4^ cell per well.

Transfection was carried out when the cell confluence reached roughly 70%. Briefly, lipofectamine RNAiMAX reagent (Termo Fisher Scientific, San Francisco Bay Area, CA, USA) was used for transfection, according to the manufacturer’s instructions. Twenty-five pmol, 5 pmol, and 1 pmol of siRNA/scRNA solutions dissolved in Opti-MEM medium (Thermo Fisher Scientific) were prepared in order to transfect 6-, 24-, and 96-well plates, respectively, at 37 °C and in the presence of CO_2_. After 24 h, transfected and control cells were treated with Meth 50 μM and NE 5 nM, which were added into the same culture medium used for transfection for a further 24 h. The silencing efficiency of β2-ARs was evaluated by Western blot.

### 4.4. Light Microscopy

#### 4.4.1. Trypan Blue in Suspended Cells 

TB staining was used to assess the percentage of dying cells. For TB, PC12 cells were seeded at a density of 10^4^ cells/well and placed within 24-well plates in 1 mL of culture medium 24 h before treatment. Seventy-two hours after Meth treatments, PC12 cells were collected and centrifuged at 800× *g* for 5 min. The cell pellet was suspended in 0.5 mL of the original culture medium to obtain a dense cell suspension, and 25 μL of cell suspension were added to a solution of 1% TB (62.5 μL) and PBS (37.5 μL). Then, cells were incubated at room temperature for 10 min. Ten μL aliquot of this solution was analyzed at light microscopy using a Bürker chamber of 10 μL volume; viable and nonviable cells were counted, and cell death was expressed as percentage of TB frankly positive cells out of the total cells. The values represent the means of three chamber counts, which were replicated for three independent experiments. Counts were carried out by investigators unaware of the treatments.

#### 4.4.2. Histochemistry and Histofluorescence in Layered Cell Pellets

For H&E and FJB experiments, 5 × 10^4^ PC12 cells were seeded on poly-lysine cover slips, which were placed in 24-well plates in a final volume of 1 mL/well.

The cell layers were stained according to H&E histochemistry and FJB histofluorescence methods.

For H&E staining, layered PC12 cells were fixed in a 4% paraformaldehyde phosphate-buffered solution (PBS) for 15 min and subsequently washed in PBS immersed in hematoxylin solution (Sigma) for some min. Hematoxylin staining was stopped by washing the slides in distilled water and plunging within the eosin solution (Sigma). After repeated washing to remove the excess of dye, cells were dehydrated in increasing alcohol solutions, clarified in xylene, and finally were covered with DPX mounting medium (Sigma). Then, slides were observed under a Nikon Eclipse 80i light microscope (Nikon, Tokyo, Japan). Cell count was performed at light microscopy at 20× magnification; for each experimental group, the number of stained cells detectable after each specific treatment was counted and expressed as a percentage of the control group. Values analyzed represent the mean percentage ± SEM for each experimental group.

For Fluoro-Jade B (FJB) (a fluorescein derivative used for histochemical staining of degenerating neurons, [83], PC12 cells were washed in PBS and fixed with paraformaldehyde 4% for 5 min, incubated with 0.06% potassium permanganate for 10 min at room temperature, and then washed in distilled water. Then, cells were incubated with 0.0004% FJB (Merck Millipore, Billerica, MA, USA) solution (consisting in 0.01% FJB in acetic acid) at room temperature for 20 min and cover slipped with mounting medium. FJB-positive cells were analyzed at Nikon Eclipse 80i light microscopy (Nikon, Tokyo, Japan), which was equipped with a florescence lamp and a digital camera connected to the NIS Elements software for image analysis (Nikon, Tokyo, Japan). For each experimental group, the count of FJB-positive cells was carried out. In detail, the number of FJB-positive cells was counted at 20× magnification within 5 distinct microscopic fields, where only distinct, not overlapped cells were counted. Values were expressed as the mean number ± SEM for each experimental group.

All counts were carried out by investigators blind to treatments.

#### 4.4.3. Immunofluorescence

Cells were incubated overnight at 4 °C with the primary antibody solution containing the rabbit anti-caspase 3 antibody (Cell Signaling Technology, Danvers, MA, USA) diluted 1:100 in 2% NGS and PBS. Then, cells were incubated for 1 h with the anti-rabbit fluoro-phore-conjugated secondary antibodies (Alexa 488, Life Technologies) diluted 1:200 in PBS at RT. All these reactions were carried out within the well plate. After washing in PBS, slices were gently pulled out and transferred on a coverslip, mounted with the mounting medium Fluoroshield (Sigma) and were observed under fluorescence microscopy (Nikon).

### 4.5. WST-1 Assay 

For WST-1 cell viability assay, PC12 cells were seeded at the density of 10^4^ cells/well and placed within 96-well plates in 100 μL of culture medium. At the end of the treatments, cell viability was assessed using the cell proliferation reagent WST-1 (Roche Diagnostics GmbH, Mannheim, Germany) according to the manufacturer’s protocol. Briefly, 10% WST-1 (4-[3-(4-Iodophenyl)-2-(4-nitro-phenyl)-2H-5-tetrazolio]-1,3-benzene sulfonate) reagent was added to each well, and the cells were incubated for 1 h at 37 °C and 5% CO_2_. Cell viability was measured as optical density at 450 nM in a microplate reader (BioTek Instruments, Winooski, VT, USA). Data were obtained in three independent experiments and expressed as the mean percentage ± SEM (assuming control as 100% WST-1).

### 4.6. Western Blot Assay

PC12 cells were lysed in a buffer (100 mM Tris-HCl, pH 7.5, 5 M NaCl, 0.5 m EDTA, 10% SDS, 1% NP40, IGEPAL), containing protease and phosphatase inhibitors, and centrifuged at 15,000× *g* for 20 min at 4 °C. After the supernatant was collected, protein concentration was determined using a protein assay kit (Sigma). Samples with 40 μg of total proteins were solubilized, electrophoresed on a 12% sodium dodecyl sulfate (SDS) polyacrylamide gel, and transferred to the nitrocellulose membrane (Bio-Rad Laboratories, Milan, Italy). The membrane was immersed in a blocking solution (5% non-fat dried milk in 20 mM Tris and 137 mM NaCl at pH 7.6 containing 0.05% Tween-20) for 2 h on a plate shaker. Subsequently, the membrane was incubated overnight at 4 °C on the plate shaker with the following primary antibodies: rabbit anti-LC3-I/LC3-II (1:1000; Cell Signaling), rabbit anti-p62 (1:1000; Sigma), and rabbit anti-β2-AR primary antibody (1:2000, Bioss Antibodies Inc., Woburn, MA, USA). To check for equal loading of the gel, membranes were probed with mouse anti-β-actin (1:5000, Sigma) or mouse anti-GAPDH (1:2000, Sigma) primary antibody. Blot was probed with horseradish peroxidase-labeled anti-mouse and anti-rabbit secondary antibody (1:2500; Calbiochem, Milan, Italy), and the bands were visualized with enhanced chemiluminescence reagents (Bio-Rad Laboratories). Image analysis was carried out by ChemiDoc System (Bio-Rad Laboratories, Milan, Italy). The intensity of the blotting was measured using the software IMAGE J and was normalized for the related housekeeping protein (β-actin or GAPDH).

In experiments that aimed to evaluate autophagy flux, 3 h before cell lysis, bafilomycin (Sigma) was added to the culture medium to obtain the treatment solution of 50 nM. Then, cells were lysed and proteins were electrophoretically resolved as previously reported [84]. Then, separated proteins were electro-transferred onto nitrocellulose membranes (Schleicher & Schuell, Dassel, Germany) by a semi-dry system (Novablot, Pharmacia Biotech, Cologno Monzese, Milano, Italy). Membranes were blocked with 3% non-fat milk in PBS and then incubated (overnight at 4 °C) with anti-LC3-I and LC3-II (1:1000, Sigma L7543). After extensive washing with PBS containing 0.1% Tween-20 (TBST), blots were incubated with 1:10,000 dilution of horseradish-peroxidase-conjugated secondary antibody (Bio-Rad Laboratories) for 1 h at room temperature. To check for equal loading of the gel, membranes were probed with mouse anti-β-actin (1:5000, Sigma A5441). Immunopositive bands were detected with a chemiluminescence detection system (GE Healthcare Biosciences, Piscataway, NJ, United States). Densitometric analysis was performed with the Quantity One software (Bio-Rad Laboratories).

Values are expressed as the mean ± S.E.M. of the optical density for each experimental group, which were calculated in three or four independent experiments. 

### 4.7. TEM and Immunocytochemistry 

For TEM, PC12 cells were centrifuged at 1000× *g* for 5 min. After removal of the supernatant, the pellet was rinsed in PBS before being fixed with a solution containing 2.0% paraformaldehyde and 0.1% glutaraldehyde in 0.1M PBS (pH 7.4) for 90 min at 4 °C, which is a fixing solution minimally covering antigen epitopes, while fairly preserving tissue architecture. After washing, specimens were postfixed in 1% OsO_4_ for 1 h at 4 °C and then dehydrated in ethanol to be finally embedded in epoxy resin. For ultrastructural morphometry and apoptotic cell count, grids containing non-serial ultrathin sections (70–90 nM thick) were examined at TEM, at a magnification of 8000×. Several grids were analyzed to count a total number of 50 cells (number of LC3 particles) or 90 cells (apoptotic/necrotic cells) for each experimental group. Each count was repeated three times by three blind observers. 

Plain TEM was implemented by a post-embedding immunocytochemistry with primary antibodies against LC3, to explore autophagy according to the manuscript “Guidelines for the Use and Interpretation of Assays for Monitoring Autophagy (4th Edition)” [85]. 

Ultrathin sections were stained with uranyl acetate and lead citrate, and they were finally examined using a JEOL JEM-100SX transmission electron microscope (JEOL, Tokyo, Japan).

#### 4.7.1. Post-Embedding Immunocytochemistry

Fixing and post-fixing solutions as well as epoxy resin were validated in previous studies for immunogold-based ultrastructural morphometry [30,86,87]. 

A post-embedding procedure was carried out on ultrathin sections collected on nickel grids, which were incubated on droplets of aqueous sodium metaperiodate (NaIO_4_), for 30 min, at room temperature to remove OsO_4_. NaIO_4_ is an oxidizing agent allowing a closer contact between antibodies and antigens by removing OsO_4_ [86].

This step improves the visualization of immunogold particles specifically placed within a sharp context of cell integrity, and it allows counting molecules within specific cell compartments [30,32,87]. Grids were washed in PBS and incubated in a blocking solution containing 10% goat serum and 0.2% saponin for 20 min at room temperature; then, they were incubated with a primary antibody solution containing rabbit anti-LC3 (Abcam, Cambridge, UK, diluted 1:50) or rabbit anti-β2AR (1:50, Bioss Antibodies Inc.) with 0.2% saponin and 1% goat serum in a humidified chamber overnight at 4°C. After washing in PBS, grids were incubated with secondary anti-rabbit antibodies conjugated with gold particles (20 nM mean diameter, BB International, Crumlin, UK), which were diluted 1:20 in PBS containing 0.2% saponin and 1% goat serum for 1 h, at room temperature. Control sections were incubated with secondary antibody only. After washing in PBS, grids were incubated on droplets of 1% glutaraldehyde for 3 min; additional extensive washing of grids with droplets of distilled water was carried out to remove an excess of salt traces and prevent the precipitation of uranyl acetate.

#### 4.7.2. Ultrastructural Morphometry

TEM analysis was performed at a magnification of 8000× [30,88], which allows the concomitant visualization of immunogold particles and all cell organelles. In order to scan the whole cell pellet within each grid square, counts were started from a corner of a randomly identified grid square. As described in detail in Lenzi et al. (2016) [89], in order to assess intracellular vacuoles and measure immunogold particles, first, we counted within each cell the number of autophagy-like vacuoles, which are vacuoles with single, double, or multiple membranes possessing the same electron density of the surrounding cytoplasm or containing some electron dense structure. For each cell, counts included the following: the total number of immunogold LC3 particles; the number of LC3-positive vacuoles; the number of LC3 immunogold particles within vacuoles and within cytosol, and the ratio between these two latter values. Data are reported as the mean ± SEM per cell from 50 cells per group. The count of the number of immunogold β2-AR particles placed on plasma membrane or within the cytosol were carried out in a total of 30 cells per group and expressed as the mean ± SEM per cell.

### 4.8. Statistical Analysis

Statistical analysis was carried out by StatView software.

For cell viability experiments, WST-1 activity was expressed as mean percentage ± SEM optical density (assuming control as 100% WST-1) calculated in three independent experiments. Values obtained in TB experiments were expressed as percentage of TB-positive cells ± S.E.M. counted in three independent experiments. For H&E, the number of stained cells detectable after each specific treatment was expressed as the mean percentage ± SEM of six independent cell counts carried out at 20× magnification within 5 distinct microscopic fields. The number of FJB-positive cells was counted in three independent experiments at 20× magnification within 5 distinct microscopic fields. Values are expressed as the mean number ± S.E.M. of FJB-positive cells.

For Western blot assay, optical density values measuring the blotting intensity were expressed as the mean percentage ± SEM (assuming control as 100%) calculated in three or four independent experiments.

For flow cytometry assay, data are expressed as the mean ± S.E.M. of four independent experiments.

Data collected for ultrastructural morphometry were expressed as an absolute number concerning: (i) autophagy-like vacuoles, (ii) LC3-positive vacuoles, (iii) LC3-immunogold particles. Furthermore, we used ratios to express the number of LC3 immunogold particles within vacuoles out of the number of cytoplasmic LC3 immuno-gold particles. Data are reported as the mean ± SEM per cell from 50 cells per group.

B2-ARs immunogold particles were counted within the cytoplasm and plasma membrane in a total of 30 cells per group. Apoptotic and necrotic cell death was counted within 90 cells (apoptotic/necrotic cells) for each experimental group and expressed as the mean percentage ± S.E.M. out of the total cells counted.

Comparisons among different groups were carried out by one-way analysis of variance (ANOVA), followed by Scheffè’s post hoc analysis. The null hypothesis (H_0_) was rejected for *p* ≤ 0.05.

## 5. Conclusions

The present study indicates that even at the single cell level, NE continues to protect against Meth-induced toxicity and that β2-AR are key in this phenomenon. 

We showed that the administration of NE alone, at a dose that significantly attenuates cell loss induced by Meth, increases LC3 expression and autophagy-like vacuoles in PC12 cells slightly but significantly compared to basal conditions. Remarkably, when administered before Meth, NE dramatically increases the number of LC3-positive vacuoles and the ratio of LC3 between vacuoles and the cytosol. Thus, NE finely tunes the polarization of LC3 within the autophagy vacuoles in a way that counteracts the effect produced by Meth: in this way, we provide clear and specific molecular evidence for a potential mechanism of action of NE-induced protection toward Meth-induced cell pathology.

## Data Availability

The data presented in this study are available on request from the corresponding author.

## Supplementary Materials

The following are available online at https://www.mdpi.com/article/10.3390/ijms22137232/s1.
