# Supplementary material for: Norepinephrine Protects against Methamphetamine Toxicity through β2-Adrenergic Receptors Promoting LC3 Compartmentalization
Source: Int J Mol Sci. 2021 Jul 5;22(13):7232. doi: 10.3390/ijms22137232 (PMC8269332; doi:10.3390/ijms22137232)
Supplement: Supplementary file 1 [file ijms-22-07232-s001.zip › ijms-1282719-supplementary.pdf]

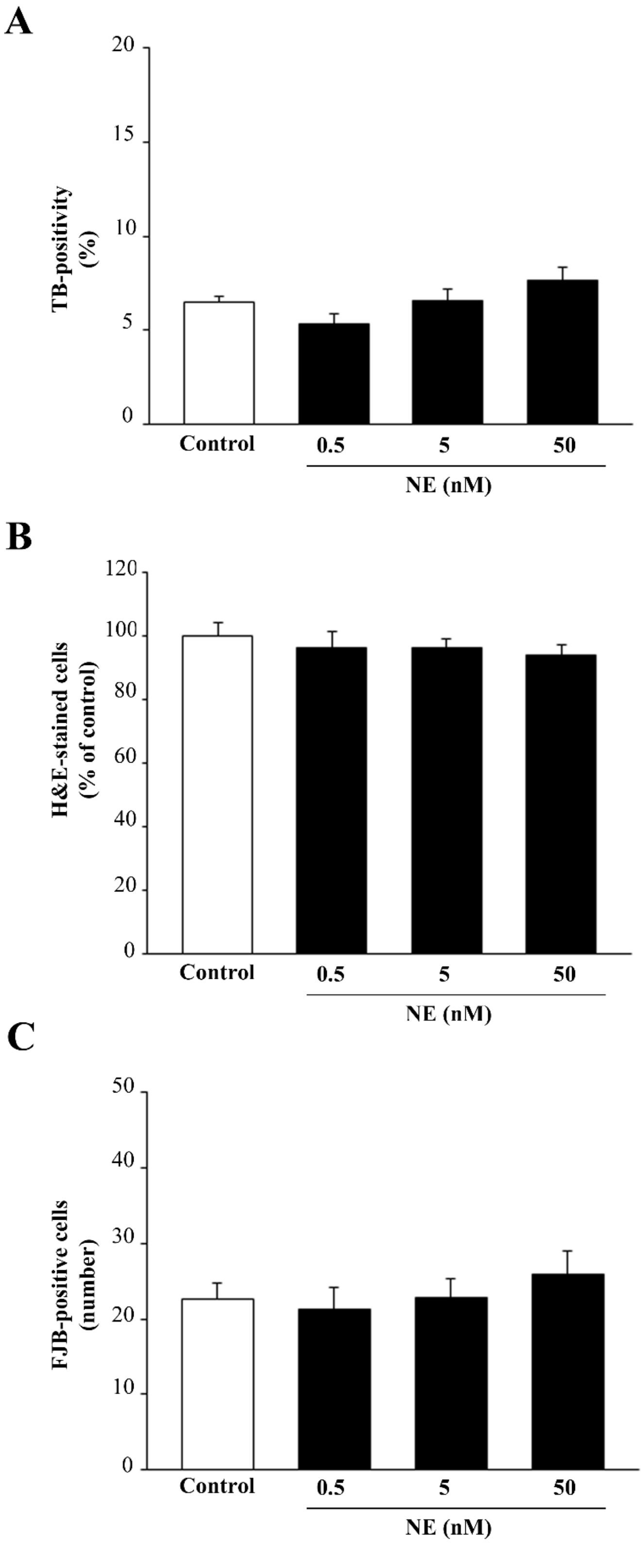

Supplementary Figure S1. Lack of toxicity in a low dose-response curve for NE. Three doses of NE (0.5 nM, 5 nM, 50 nM) were administered for 72 hours to PC12 cells. The effects on cell viability were assessed by TB staining (**A**); H&E staining (**B**); FJB histofluorescence (**C**). Any change in cell viability compared with controls was detected for each dose of NE by using any staining technique. Data are given as the mean+SEM of 9 independent counts for TB; 6 independent counts for H&E and FJB. Inferential statistics was carried out with ANOVA with Scheffè's post-hoc analysis (DF=3).

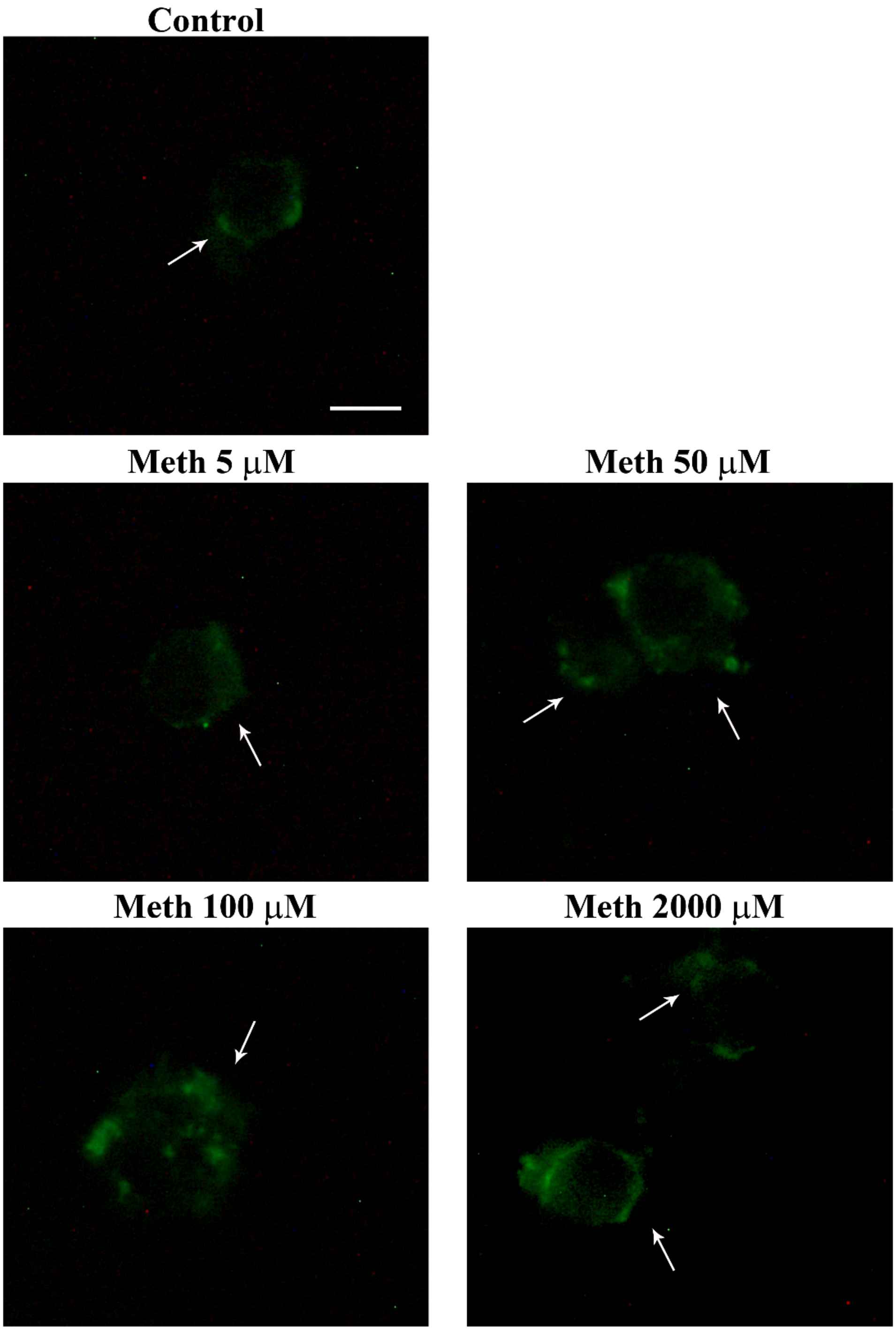

Supplementary Figure S2. Meth does not increases the caspase 3-immunofluorescence. (**A**) Representative pictures of caspase 3-immunofluorescent cells observed after exposure to increasing doses of Meth (from 5 μM up to 2000 μM). Arrows indicate the caspase-3-immunofluorescent cells. Scale bar = 6 μm.

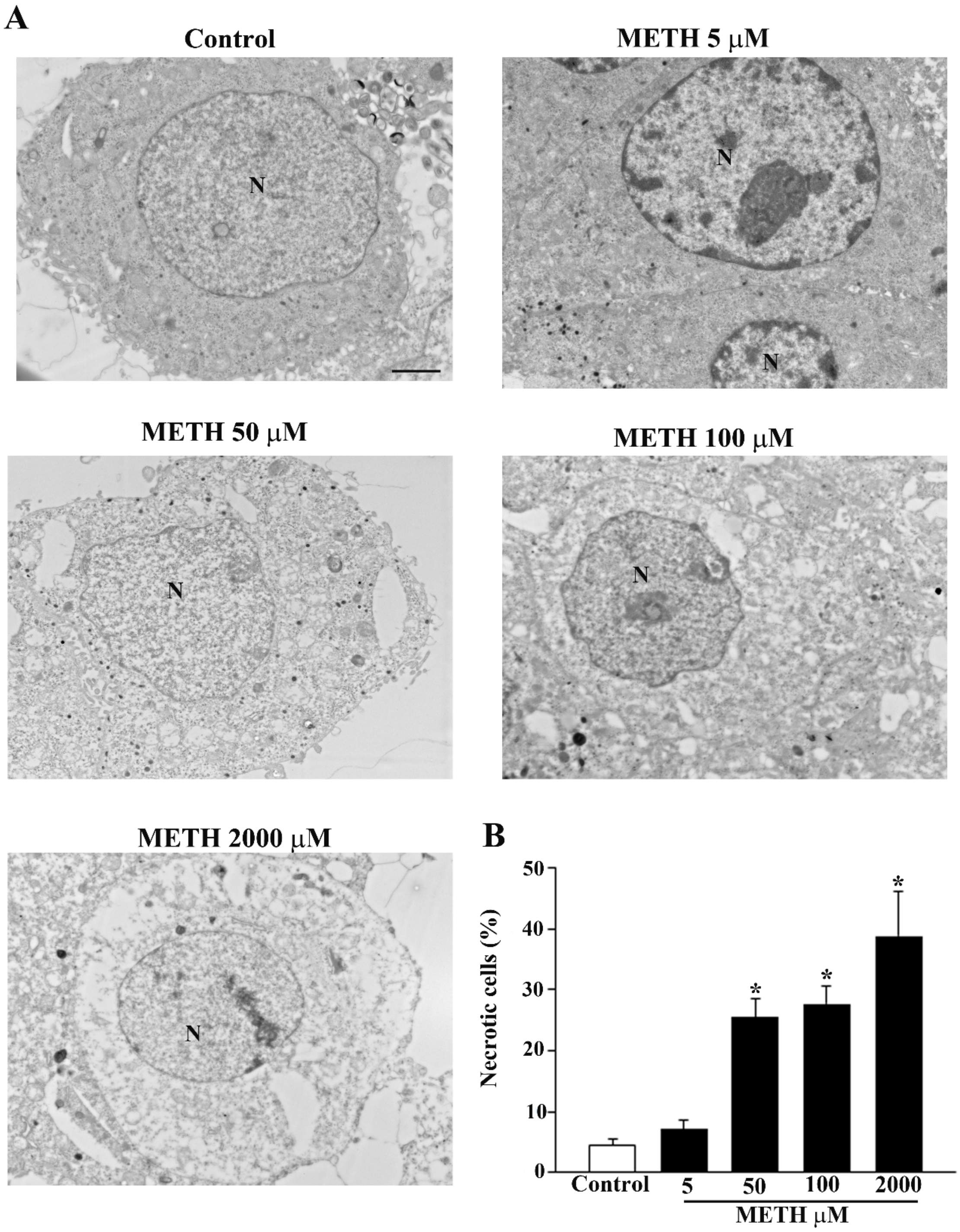

Supplementary Figure S3. Meth induces necrotic cell death. (**A**) Representative micrographs of PC12 cells after increasing doses of Meth (from 5 μM up to 2000 μM). (DF=4). Scale bar = 1 μm.

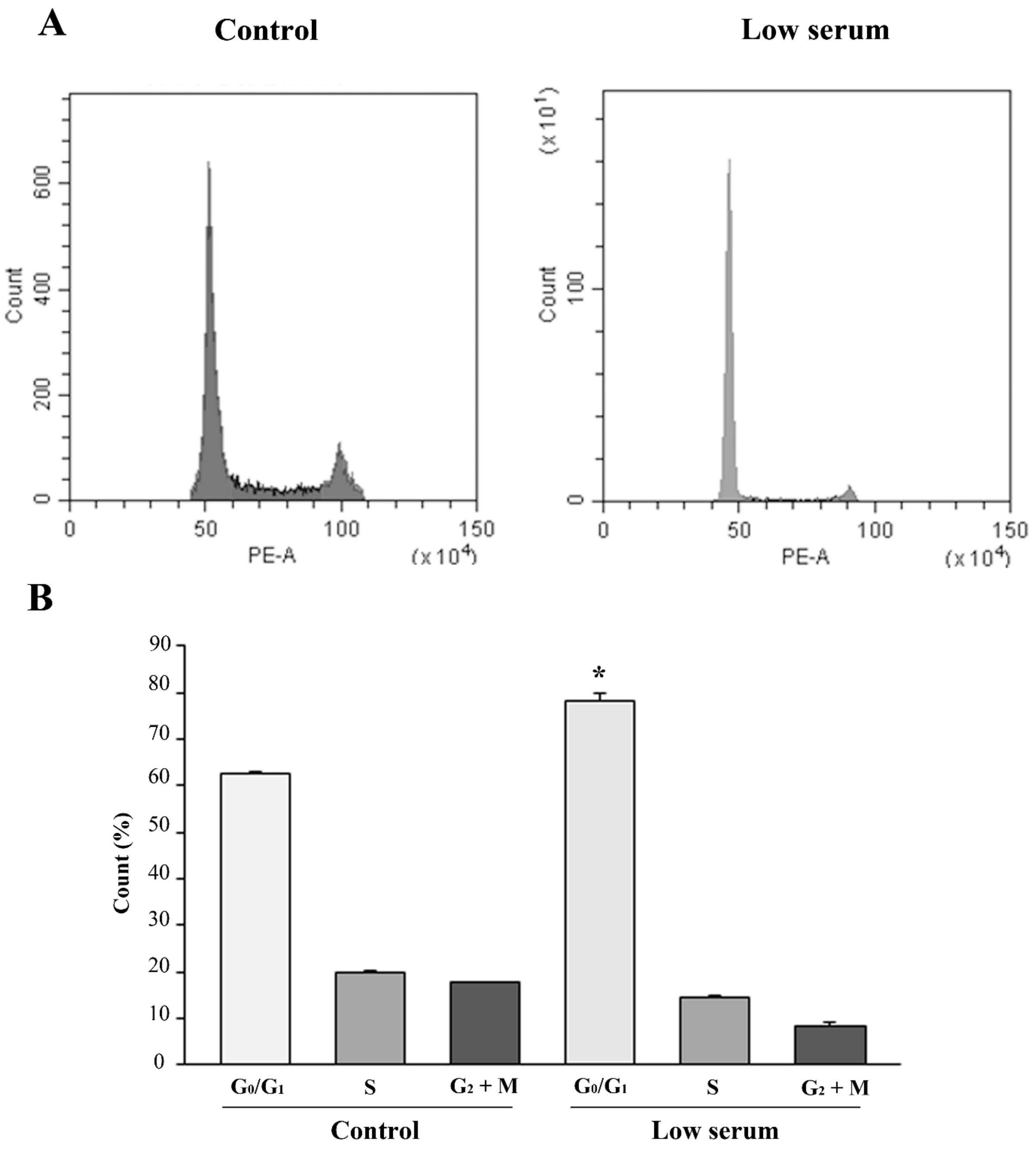

Supplementary Figure S4. Cell cycle analysis of PC12 cells under standard or low serum culture conditions (starvation). Starvation synchronizes PC12 cells by significantly increasing the percentage of cells in the G_0/G_1 phase of the cell cycle. (DF=5) *P<0.05 compared with all other groups.

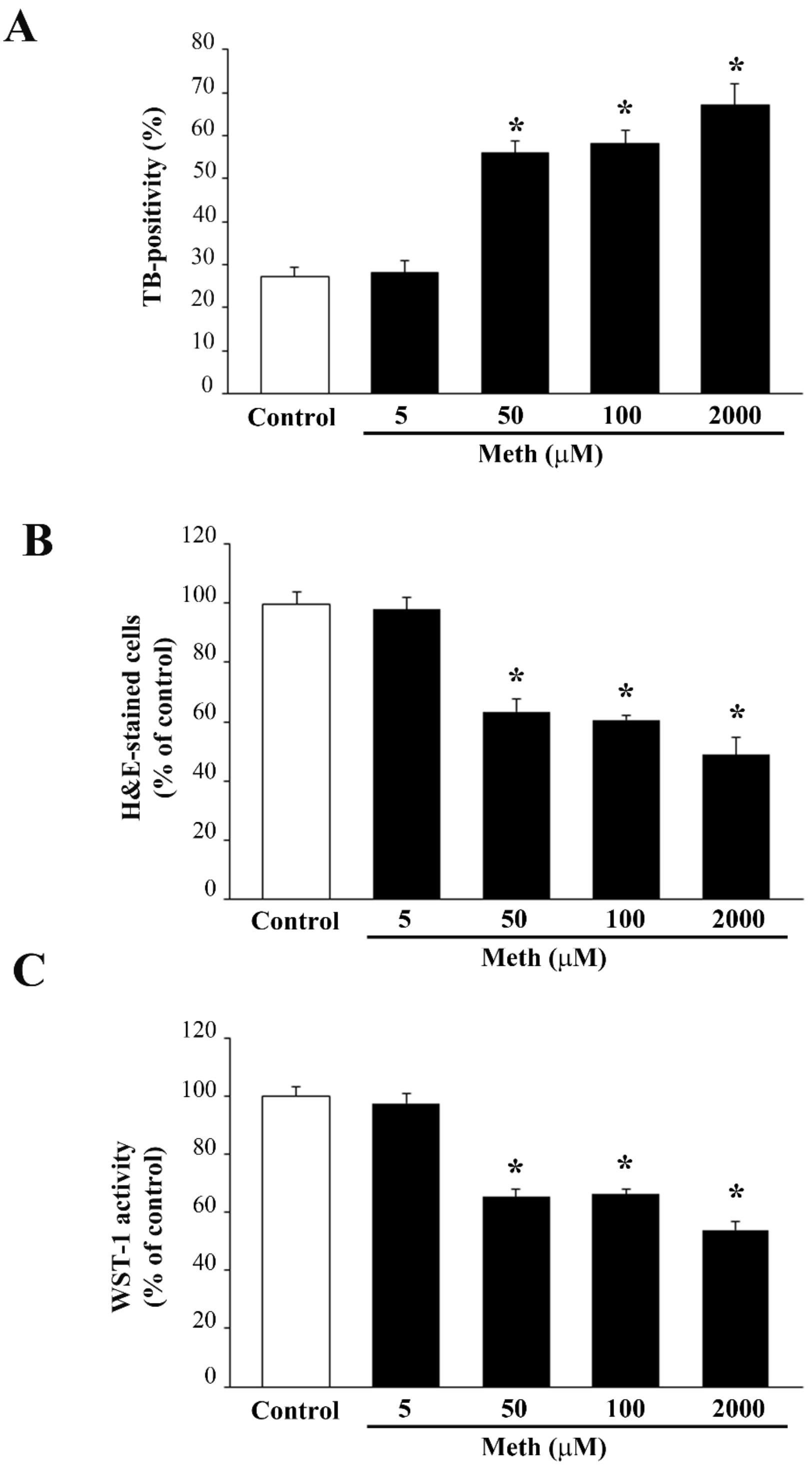

Supplementary Figure S5. The dose response curve for Meth-induced toxicity is not modified in synchronized PC12 cells. Cell viability of PC12 cells treated with increasing doses of Meth (from 5 μM up to 2000 μM) is assessed by (**A**) TB, (**B**) H&E, (**C**) WST-1 assay. (DF=4). *P<0.05 compared with control.

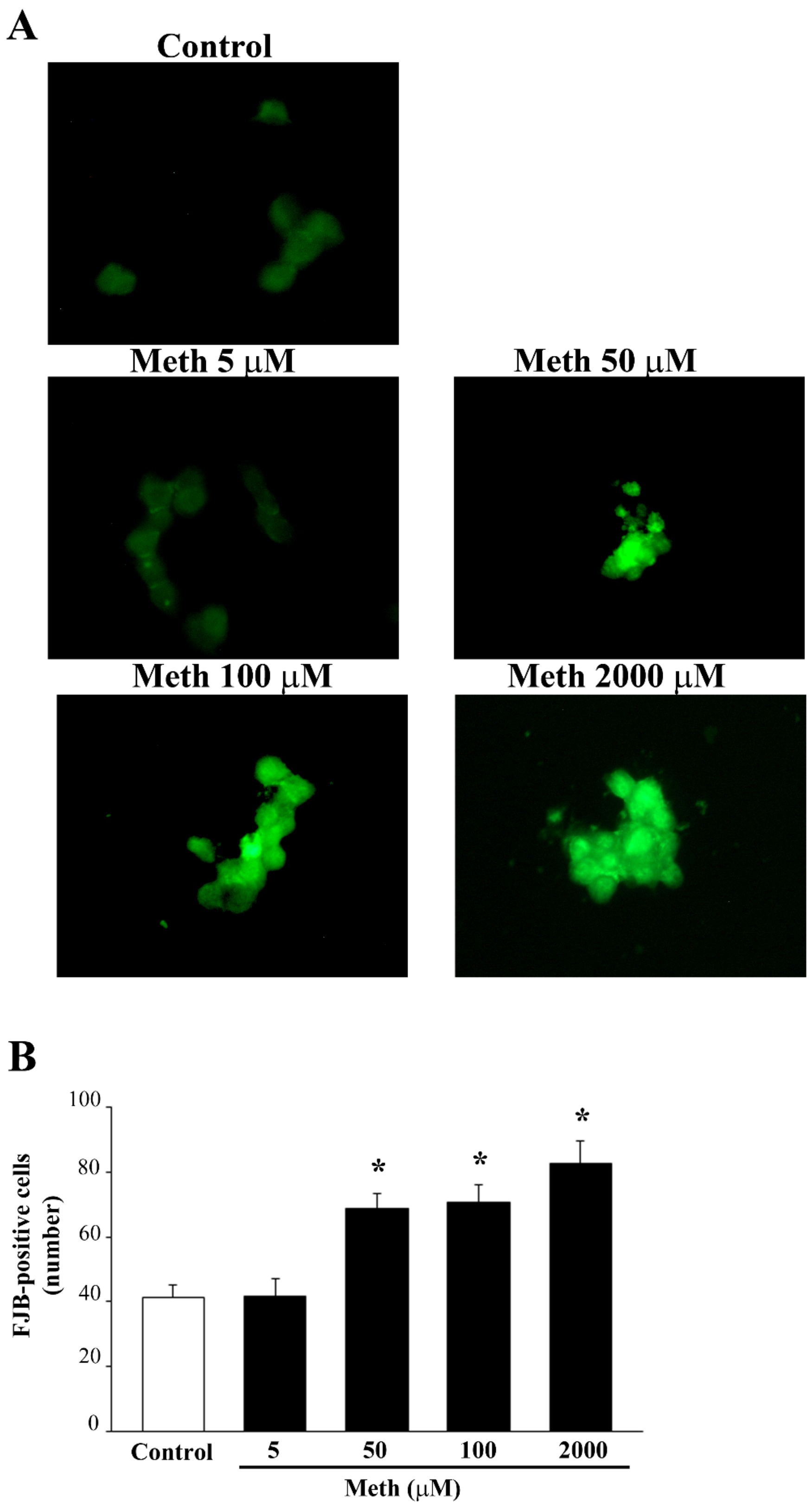

Supplementary Figure S6. FJB fluorescence in synchronized PC12 cells after increasing doses of Meth. (**A**) Representative pictures of FJB-fluorescent cells observed after exposure to increasing doses of Meth (from 5 μM up to 2000 μM). The related number of FJB-fluorescent cells is reported in the graph (**B**). (DF=4) *P<0.05 compared with control.

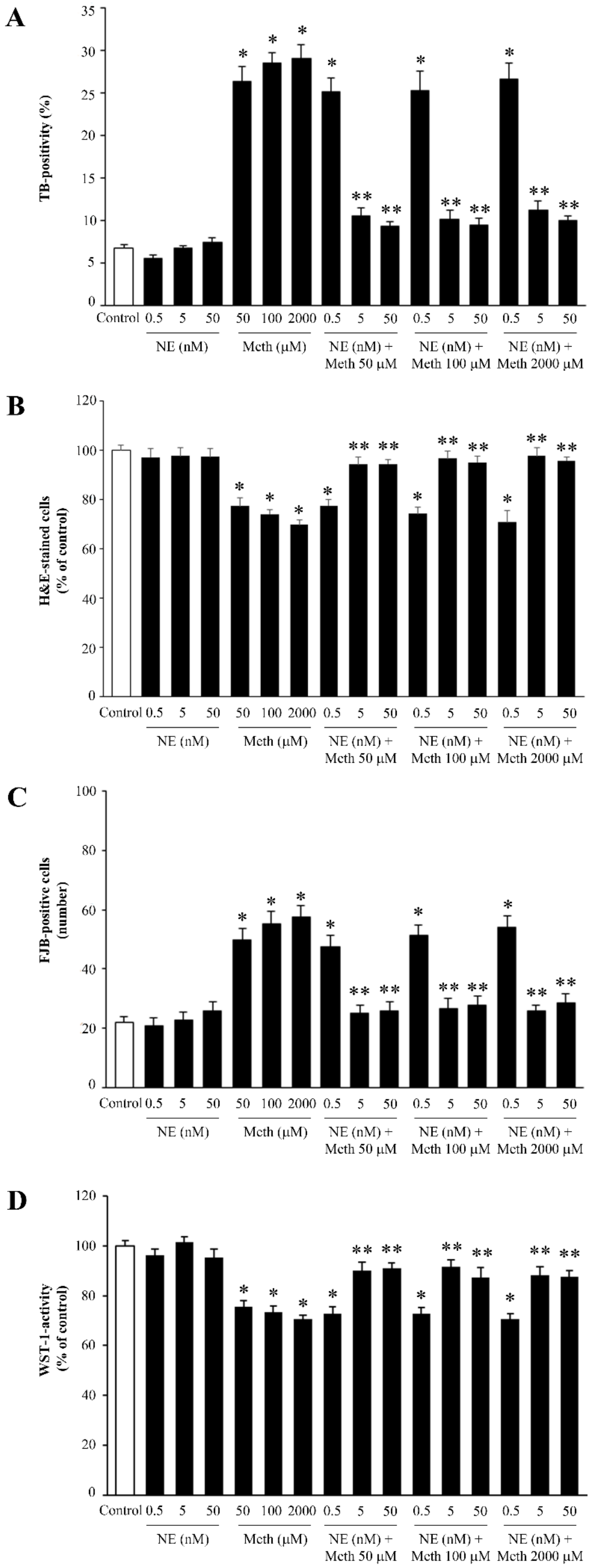

Supplementary Figure S7. Dose-response of NE-induced protection against Meth-induced toxicity. Three different concentrations of NE (0.5 nM, 5 nM, 50 nM) were administered 30 min before saline (Controls) or various toxic doses of Meth (50 µM, 100 µM, 1 mM, 2 mM). Norepinephrine at the lowest dose (50 µM) fully protects against Meth toxicity even for the high Meth dose (2 mM). (**A**) Graph for TB-stained cells; (**B**) graph of H&E-stained cells; (**C**) graph of FJB-stained cells; (**D**) graph of WST-1 viability assay. Data are given as the mean+SEM of 9 independent counts for TB and WST-1; 6

independent counts for H&E and FJB. Inferential statistics was carried out with ANOVA with Scheffè's post-hoc analysis. (DF=15) *$P<0.05$ compared with controls. **$P\leq$ 0 05 compared with Meth.

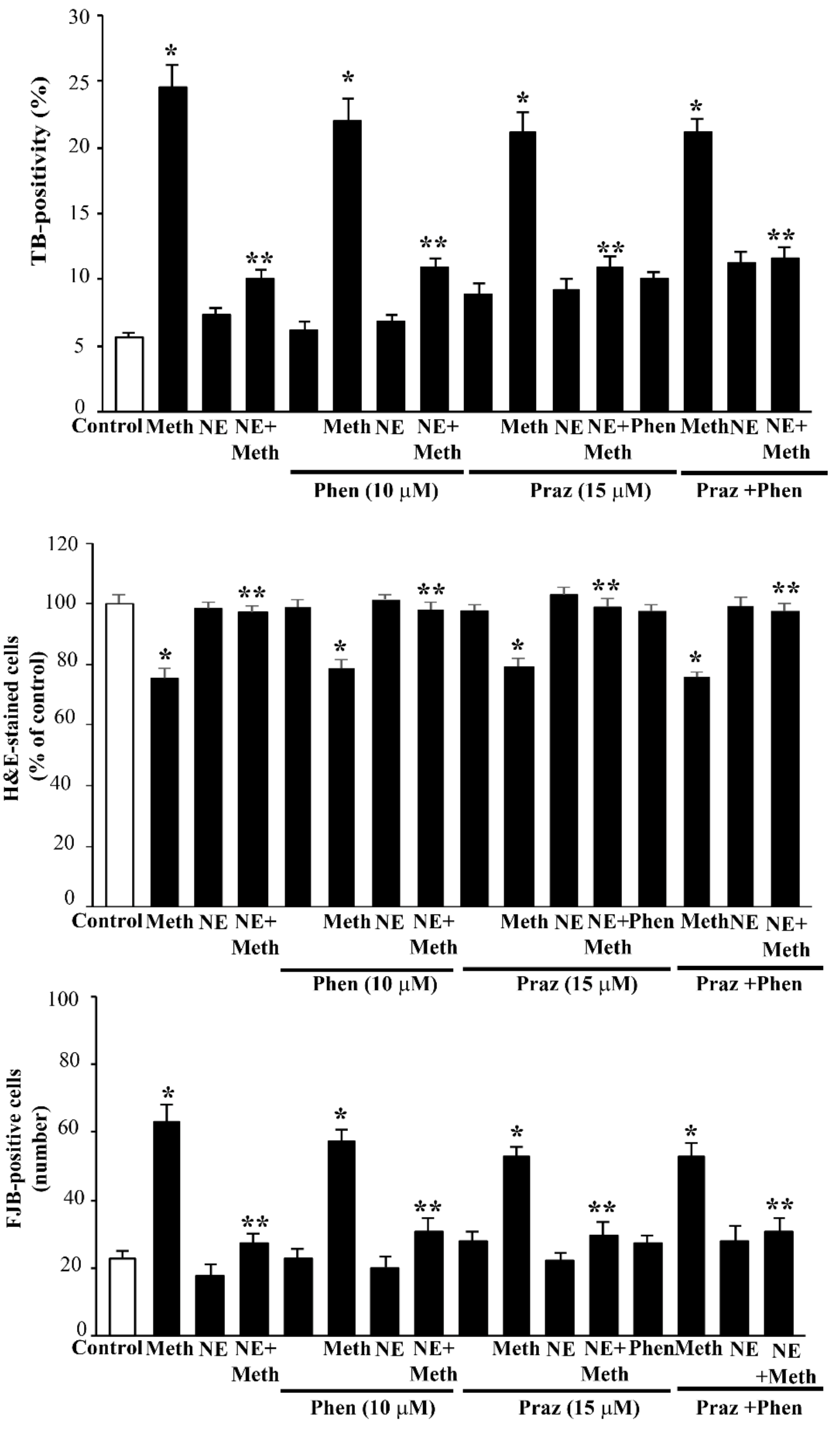

Supplementary Figure S8. Pre-administration of neither the α1-AR agonist (phenylephrine) nor the α1-AR antagonist (prazosin) alters Meth toxicity. The α1-AR agonist phenylephrine ("Phen", 10 μM) and the α1-AR antagonist prazosin ("Praz", 15 μM) do not alter Meth (50 μM) toxicity. In the combined treatment groups, NE (5 nM) and/or phenylephrine were administered 30 min before Meth administration; prazosin was administered 15 min before NE in the group "NE+Praz+Meth", or 45 min before Meth in the group "Praz+Meth". Phenylephrine was administered 30 min before Meth in the group "Phen+Meth" and "Phen+NE+Meth". PC12 cells were stained at 72 hours after Meth. (**A**) Graph reporting TB staining; (**B**) graph reporting H&E staining (**C**) graph reporting FJB staining. Data are given as the mean+SEM of 9 independent counts for TB; 6 independent counts for H&E and FJB. Inferential statistics was carried out with ANOVA with Scheffè's post-hoc analysis. (DF=15) *P<0.05 compared with controls. **P≤ 0 05 compared with Meth.

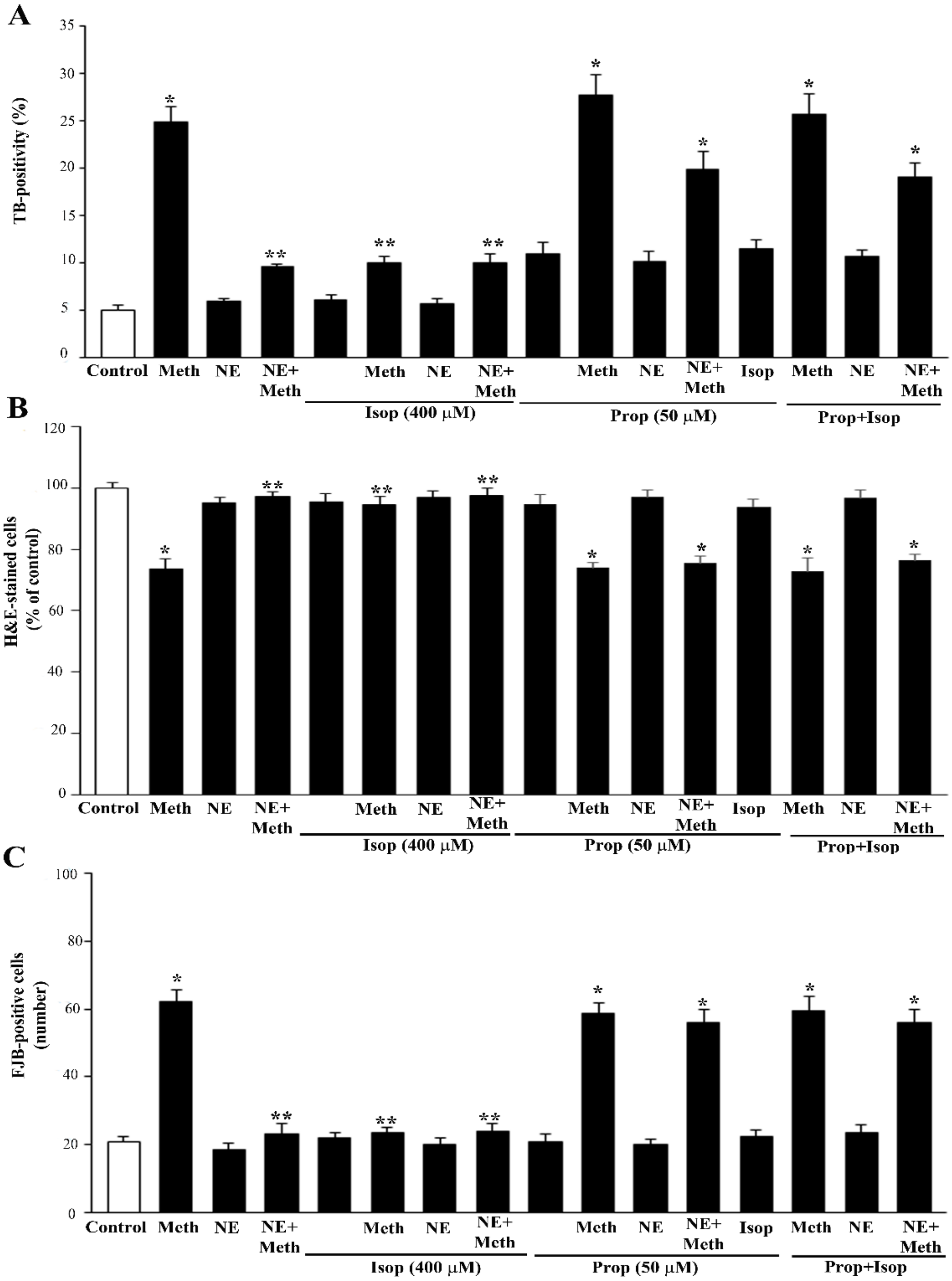

Supplementary Figure S9. Pre-administration of the ⚜-AR agonist (isoproterenol) protects against Meth toxicity. This effect is occluded by the ◎1-AR an-tagonist (propranolol).The non-selective β-AR agonist isoproterenol ("Isop", 400 μM) fully protects against the toxicity induced by Meth (50 μM). This protective effect is occluded in the presence of the non-selective β-AR antagonist propranolol ("Prop", 50 μM). Propranolol also fully antagonize NE (5 nM) induced protection against Meth toxicity, while it does not significantly mod-ify Meth toxicity or spontaneous toxicity ongoing in control cells when administered alone. In the combined treatments NE and isoproterenol were administered 30 min before Meth administration; propranolol was administered 15 min before NE in the group "NE+Prop+Meth", or 45 min before Meth in the group "Prop+Meth". PC12 cells were stained at 72 hours after Meth. (A) Graph reporting TB staining; (B) graph reporting H&E staining; (C) graph reporting FJB staining. Data are given as the mean+SEM of 9 independent counts for TB; 6 independent counts for H&E and FJB. Inferential statistics was carried out with ANOVA with Scheffè's post-hoc analysis. (DF=15). *P<0.05 compared with controls. **P≤ 0 05 compared with Meth.

A

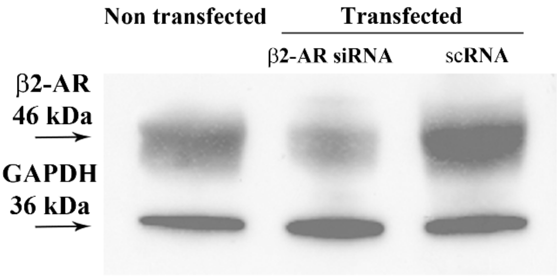

B

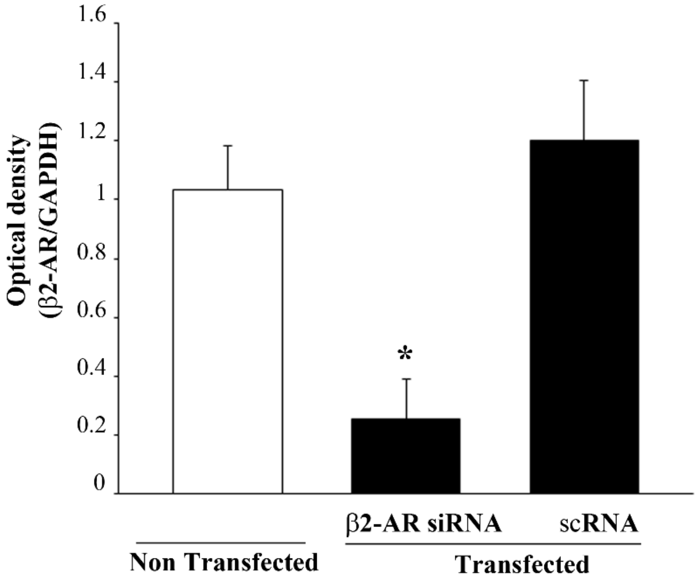

Supplementary Figure S10. Transfection with β2-AR siRNA silences the expression of β2-AR in PC12 cells. (**A**) Western blot for β2-AR in transfected and non transfected cells. (**B**) Optical density shows that the expression of β2-AR is reduced over than 75% within cells transfected with β2-AR siRNA. .*P<0.05 compared with control and scRNA transfected cells. (DF=2).

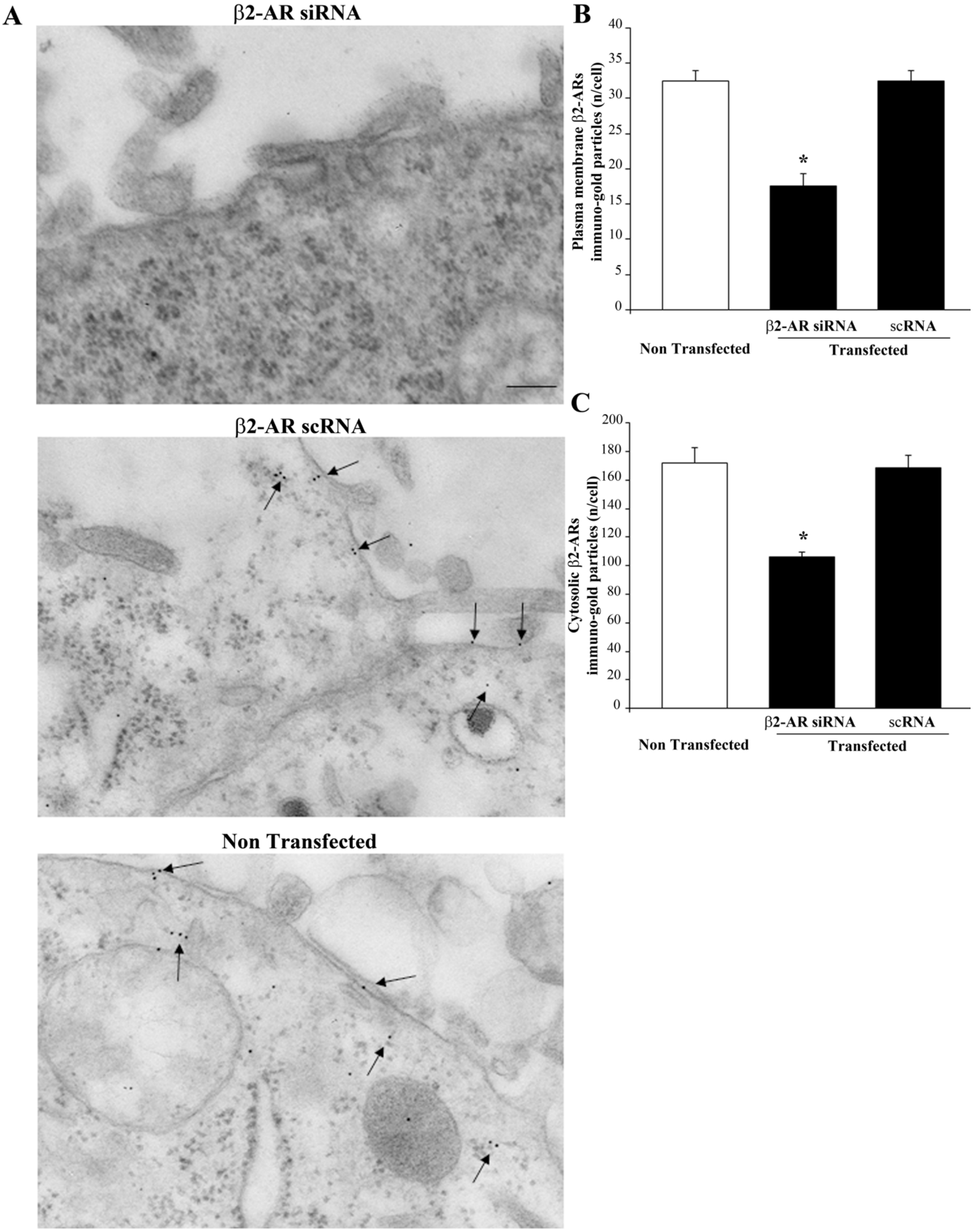

Supplementary Figure S11. Transfection with β2-AR siRNA removes β2-AR from the plasma membrane (**A**) Representative TEM micrographs show the lack of β2-AR on plasma membrane of β2-AR siRNA transfected cells. The graphs report the number of β2-AR-related immune-gold particles per cell which were counted on the plasma membrane (**B**) and within the cytosol (**C**) of transfected and non-transfected cells. Arrows indicate anti-β2-AR immunogold particles adherent to the plasma membrane and into the cytosol.*$P<0.05$ compared with control and β2-AR scRNA transfected cells. (DF=2) Scale bar = 100 nm.
